# EphB2 Signaling Is Implicated in Astrocyte-Mediated Parvalbumin Inhibitory Synapse Development

**DOI:** 10.1523/JNEUROSCI.0154-24.2024

**Published:** 2024-09-26

**Authors:** Samantha N. Sutley-Koury, Christopher Taitano-Johnson, Anna O. Kulinich, Nadia Farooq, Victoria A. Wagner, Marissa Robles, Peter W. Hickmott, Vijayalakshmi Santhakumar, Patrice N. Mimche, Iryna M. Ethell

**Affiliations:** ^1^Division of Biomedical Sciences and Biomedical Sciences Graduate Program, School of Medicine, University of California Riverside, Riverside, California 92521; ^2^Neuroscience Graduate Program, University of California Riverside, Riverside, California 92521; ^3^Department of Dermatology, and Department of Medicine, Division of Gastroenterology and Hepatology, Indiana University School of Medicine, Indianapolis Indiana 46202

**Keywords:** astrocyte, EphB receptor, hippocampus, inhibition, parvalbumin, synapse

## Abstract

Impaired inhibitory synapse development is suggested to drive neuronal hyperactivity in autism spectrum disorders (ASD) and epilepsy. We propose a novel mechanism by which astrocytes control the development of parvalbumin (PV)-specific inhibitory synapses in the hippocampus, implicating ephrin-B/EphB signaling. Here, we utilize genetic approaches to assess functional and structural connectivity between PV and pyramidal cells (PCs) through whole-cell patch–clamp electrophysiology, optogenetics, immunohistochemical analysis, and behaviors in male and female mice. While inhibitory synapse development is adversely affected by PV-specific expression of EphB2, a strong candidate ASD risk gene, astrocytic ephrin-B1 facilitates PV→PC connectivity through a mechanism involving EphB signaling in PV boutons. In contrast, the loss of astrocytic ephrin-B1 reduces PV→PC connectivity and inhibition, resulting in increased seizure susceptibility and an ASD-like phenotype. Our findings underscore the crucial role of astrocytes in regulating inhibitory circuit development and discover a new role of EphB2 receptors in PV-specific inhibitory synapse development.

## Significance Statement

The findings presented in this study describe a novel mechanism by which astrocytes regulate the establishment of connections between parvalbumin (PV) interneurons and pyramidal neurons. We also present new evidence showing the role of presynaptic EphB2 in the formation of inhibitory synapses, specifically between PV-expressing interneurons and pyramidal neurons. Impaired inhibition is suggested to underlie the development of neuronal hyperactivity in several neurodevelopmental disorders (NDDs), and EphB2 receptor itself is also implicated in the pathogenesis of autism. Therefore, this study not only addresses critical gaps in our understanding but also offers clinical relevance as EphB2 signaling in PV interneurons may be a promising therapeutic target to correct inhibitory circuit dysfunction in NDDs.

## Introduction

Astrocytes critically regulate many aspects of synaptic physiology, including synaptogenesis, synapse elimination, synaptic transmission, and synapse plasticity through both contact-dependent mechanisms and release of soluble factors (Allen and Eroglu, 2017; Augusto-Oliveira et al., 2020). Although several mechanisms by which astrocytes regulate excitatory synapse development and function have been described, our knowledge of how astrocytes regulate inhibitory synapse development and function remains limited (Clarke and Barres, 2013; Allen and Eroglu, 2017; Augusto-Oliveira et al., 2020). Astrocytes are shown to promote the development of inhibitory synapses, increasing the number of presynaptically active GABAergic synapses and the frequency of miniature inhibitory postsynaptic current (mIPSCs) in mixed cultures (Elmariah et al., 2005; Hughes et al., 2010). Additionally, the strength of GABA_A_ receptor (GABA_A_R)-mediated currents is potentiated by astrocytes in cultured hippocampal neurons, showing enhanced GABA-mediated currents only in neuronal somata contacting astrocytes (Liu et al., 1996). Furthermore, our previous findings show that astrocytic ephrin-B1 negatively regulates excitatory synapse formation yet enhances inhibition in the developing but not adult CA1 hippocampus through an undiscovered mechanism (Koeppen et al., 2018; Nguyen et al., 2020). We also showed that the density of parvalbumin (PV)-expressing cells was decreased in the developing hippocampus of astrocyte-specific ephrin-B1 KO mice, suggesting a potential role of ephrin-B signaling in PV cell development (Nguyen et al., 2020). However, it was not clear whether the changes we observed were a result of altered signaling between astrocytic ephrin-B1 and its EphB receptors in PV cells or indirect effects due to changes in astrocyte phenotype. In this study, we hypothesized that the effects of astrocytic ephrin-B1 on PV→pyramidal cell (PC) connectivity in the developing hippocampus are mediated through EphB2 receptor signaling in PV cells.

Ephrin-B/EphB signaling is well studied in excitatory synapses (Lim et al., 2008; Pasquale, 2008; Klein, 2012; Sheffler-Collins and Dalva, 2012), including their role in dendritic spine formation and maturation (Ethell et al., 2001; Moeller et al., 2006), AMPAR phosphorylation and trafficking to the synapse (Kayser et al., 2006; Hussain et al., 2015), NMDAR localization and function (Takasu et al., 2002; Nolt et al., 2011), as well as regulation of LTP (Henderson et al., 2001; Contractor et al., 2002). However, little is known about the mechanism of ephrin-B/EphB signaling in inhibitory synapse development or how astrocytes influence inhibitory synapse development through ephrin-B/EphB signaling. Although postsynaptic EphB receptors are known to facilitate excitatory synapse formation between axon terminals and dendrites (Henkemeyer et al., 2003; Lim et al., 2008; Pasquale, 2008; Klein, 2012; Sheffler-Collins and Dalva, 2012; Yang et al., 2013), here we propose that presynaptic EphB2 may act as a repellent, preventing innervation between the inhibitory axon terminals of PV interneurons and PCs.

Impaired inhibition and inhibitory synapse pathologies and PV interneuron hypofunction, as well as glial cell dysfunction, are common findings in many neurodevelopmental disorders (NDDs; Petrelli et al., 2016; Ali Rodriguez et al., 2018; Filice et al., 2020; Contractor et al., 2021; Tan and Eroglu, 2021). Indeed, *E*/*I* imbalance is hypothesized to drive pathology in NDDs (Rubenstein and Merzenich, 2003). More specifically, impaired inhibition is thought to underlie the development of hyperactive neuronal networks in autism spectrum disorder (ASD) and epilepsy (Sohal and Rubenstein, 2019; Tang et al., 2021). Dysfunctions in PV interneurons, including reduced density of PV interneurons, reduced PV expression, impaired PNN formation, and reduced PV cell activity, have been observed in ASD, suggesting that PV interneurons may play a critical role in regulating *E*/*I* balance and the pathogenesis of NDDs (Lawrence et al., 2010; Hashemi et al., 2016; Contractor et al., 2021).

The goal of this study was to determine whether astrocytes regulate PV→PC connectivity through ephrin-B/EphB signaling. We targeted the Postnatal Day (P)14–P28 developmental period that is critical for PV interneuron development in the hippocampus (Contractor et al., 2021) and used loss- and gain-of-function approaches to regulate ephrin-B1 levels in astrocytes and EphB2 receptor expression in PV interneurons during this period. Functional and structural PV→PC connectivity was assessed through whole-cell patch–clamp electrophysiology, optogenetics, immunohistochemical and biochemical analysis, seizure susceptibility assays, and mouse behaviors. Here we report a novel mechanism by which astrocytic ephrin-B1 regulates inhibitory synapse development, specifically PV→PC connectivity, and propose a new role of ephrin-B1/EphB2 receptor signaling in PV cells as the underlying mechanism.

## Materials and Methods

### Ethics statement

All mouse studies were done according to National Institutes of Health and Institutional Animal Care and Use Committee at the University of California Riverside guidelines; animal welfare assurance number A3439-01 is on file with the Office of Laboratory Animal Welfare. Mice were maintained in an Association for Assessment and Accreditation of Laboratory Animal Care-accredited facility under a 12 h light/dark cycle and fed standard mouse chow.

### Mouse lines

**Figure 1.**
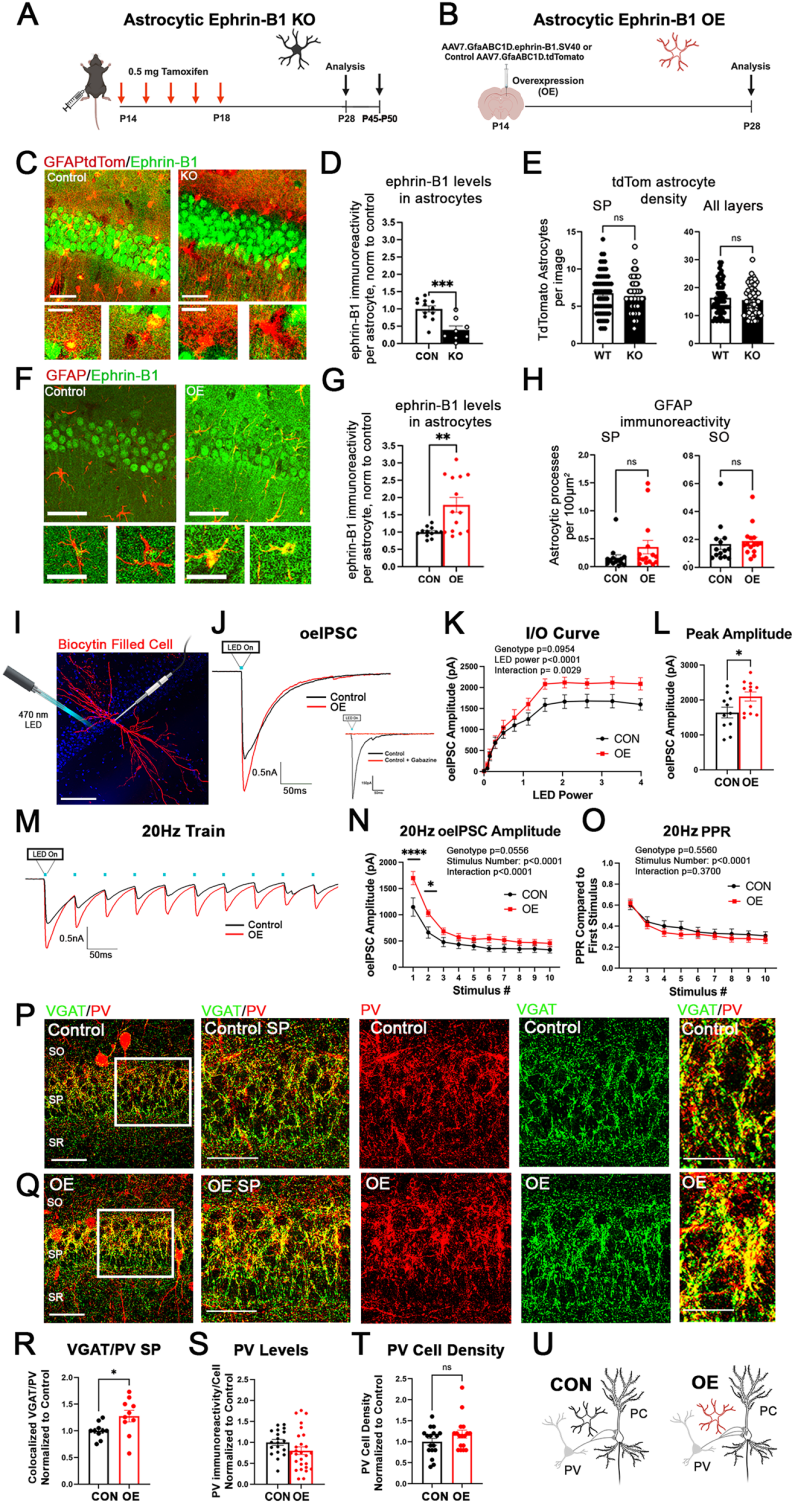
Astrocytic ephrin-B1 OE increases PV→PC connectivity. ***A***, ***B***, Graphics showing experimental timelines for astrocytic ephrin-B1 deletion (***A***) and OE (***B***). ***C***, Confocal images showing immunolabeling of ephrin-B1 (green) and TdTomato (red) in control and KO astrocytes; scale bars, 50 μm (top panels) and 25 μm (bottom panels). ***D***, Graph shows the quantification of ephrin-B1 mean fluorescence intensity in TdTomato-expressing control and KO astrocytes. Astrocytic ephrin-B1 KO significantly reduces ephrin-B1 immunoreactivity in astrocytes (8–12 images/4 mice per group; *t* test; ***p < 0.001; Extended Data Table 1-1). ***E***, Graphs show the number of TdTomato-expressing astrocytes in the CA1 hippocampus and SP layer of control and KO mice. Deletion of astrocytic ephrin-B1 does not influence the number of TdTomato-expressing astrocytes in the CA1 hippocampus (61–62 images/4 mice per group; *t* test; *p* > 0.05; Extended Data Table 1-1). ***F***, Confocal images showing immunolabeling of ephrin-B1 (green) and GFAP (red) in control and OE astrocytes; scale bars, 50 μm (top panels) and 25 μm (bottom panels). ***G***, The graph shows the quantification of ephrin-B1 mean fluorescence intensity levels of GFAP immunoreactivity in OE astrocytes normalized to the contralateral, noninjected side of the same brain slice. OE significantly increases ephrin-B1 immunoreactivity in astrocytes (11 images/3 mice per group; *t* test; **p < 0.01; Extended Data Table 1-1). Note that OE images were collected with a lower laser intensity and gain than the WT group to prevent saturation of the ephrin-B1 signal in astrocytes, which resulted in lower ephrin-B1 signal in neurons than in the KO group and their corresponding controls. ***H***, Graphs show the number of GFAP-immunoreactive processes in the SP and SO layers of CA1 hippocampus of noninjected and OE side (14–15 images/4–5 mice per group; *t* test; *p* > 0.05; Extended Data Table 1-1). ***I***, Representative image of a biocytin-filled PC in the CA1 hippocampus, labeled with streptavidin (red) following whole-cell recording. Scale bar, 100 μm. ***J***, Representative current traces of the oeIPSC recorded from PCs in hippocampal slices of OE and vehicle-injected mice (control) following the activation of PV interneurons expressing ChR2 with 400 nm LED light. Inset, The black line shows the optically evoked inhibitory currents recorded in a CA1 PC prior to application of gabazine. The red line shows the current response to optical stimulation after bath application of 10 μM gabazine. Bath application of gabazine successfully abolished oeIPCs, indicating that the currents were inhibitory. ***K***, The IO curve shows the average oeIPSC amplitude in OE and control groups plotted against LED power. There is a significant effect of LED power and interaction between genotype and LED power (11–12 cells per group, 7–8 mice per group, two-way ANOVA, Extended Data Table 1-1). ***L***, The graph shows the average peak amplitude of the last five stimulations in the IO curve. The ephrin-B1 OE group shows a significant increase in the peak oeIPSC amplitude (11–12 cells per group; 7–8 mice per group; *t* test; *p < 0.05; Extended Data Table 1-1). ***M***, Representative traces of the oeIPSCs in control and OE groups, generated during stimulation with a 20 Hz train of 10 LED pulses. ***N***, The graph shows the average oeIPSC amplitude in control and OE during each LED pulse within a 20 Hz train. There is a significant increase in the average oeIPSC amplitude in response to the first two stimuli in OE compared with the control group (8–9 cells per group; 7–8 mice per group; two-way ANOVA; *p < 0.05; ****p < 0.0001; Extended Data Table 1-1). ***O***, The graph shows the average oeIPSCs normalized to the first stimulus in the 20 Hz train to assess short-term plasticity with no significant effect of genotype or interaction (8–9 cells per group, 7–8 mice per group, two-way ANOVA, Extended Data Table 1-1). ***P***, ***Q***, Confocal images of brain slices from control (***P***) and OE (***Q***) mice immunolabeled against VGAT (green) and PV (red); scale bar, 50 μm (left panels), 25 μm (middle panels), 10 μm (right panels). ***R***, The graph shows the average number of VGAT/PV colocalized puncta in the OE group normalized to the contralateral noninjected side. The OE group shows a significant increase in VGAT/PV colocalized puncta in the SP layer of the CA1 hippocampus (10 images/5 mice per group; *t* test; *p < 0.05; **p < 0.01; ***p < 0.001; Extended Data Table 1-1). ***S***, The graph shows the PV immunoreactivity levels within PV interneurons of OE mice. PV levels were not affected by OE of astrocytic ephrin-B1 (19–27 cells/3 mice per group; *t* test; *p* > 0.05; Extended Data Table 1-1). ***T***, The graph shows density of PV-expressing cells in the CA1 hippocampus of OE mice compared with the contralateral noninjected side. OE does not significantly affect the density of PV-expressing cells in the CA1 hippocampus (17 images/4 mice per group; *t* test; *p* > 0.05; Extended Data Table 1-1). ***U***, Drawing depicts differences in PV→PC connectivity between ephrin-B1 OE and control groups. All data are represented as mean ± SEM. Graphics created with Biorender.com.

We used several mouse lines to analyze the effects of ephrin-B1 KO in astrocytes as follows: (1) ERT2-CreGFAP^+/+^ephrin-B1^flox/y^ KO or ERT2-CreGFAP^−/−^ephrin-B1^flox/y^ control mice were generated by breeding ephrin-B1^flox/flox^ or ephrin-B1^flox/y^ (129S-Efnb1 flox/+/J, RRID:IMSR_JAX:007664) mice with ERT2-CreGFAP^+/+^ or ERT2-CreGFAP^−/−^ [B6.Cg-Tg(GFAP-cre/ERT2)505Fmv/J, RRID:IMSR_JAX: 012849] mice. (2) Thy1GFP-ERT2-CreGFAP^+/+^ephrin-B1^flox/y^ KO and Thy1GFP-ERT2-CreGFAP^−/−^ephrin-B1^flox/y^ control male mice, in which GFP was expressed in excitatory cells of the hippocampus with active Thy1 promotors, were generated by crossing Thy1GFP-M mice [Tg(Thy1-EGFP)MJrs/J, RRID:IMSR_JAX:007788] with ERT2-CreGFAP^+/+^ephrin-B1^flox/y^ KO or ERT2-CreGFAP^−/−^ephrin-B1^flox/y^ control mice. (3) PV-tdTomato-CreGFAP^+/+^ephrin-B1^flox/y^ KO or PV-tdTomato ERT2-CreGFAP^−/−^ephrin-B1^flox/y^ control mice were generated by crossing C57BL/6-Tg(Pvalb-tdTomato)15Gfng/J mice (RRID:IMSR_JAX:027395) with ERT2-CreGFAP^+/+^ephrin-B1^flox/flox^ KO female or ERT2-CreGFAP^−/−^ephrin-B1^flox/flox^ female control mice. (4) Rosa-CAG-LSL-tdTomato reporter mice (CAG-tdTomato; RRID:IMSR_JAX:007909) were bred with ERT2-CreGFAP^+/+^ or ERT2-CreGFAP^−/−^[B6.Cg-Tg(GFAP-cre/ERT2)505Fmv/J RRID:IMSR_JAX:012849] mice to generate tdTomatoERT2-CreGFAP mice first. Then, tdTomatoERT2-CreGFAP male mice were crossed with ephrin-B1^flox/flox^ or with ephrin-B1^+/+^ female mice to obtain either tdTomatoERT2-CreGFAPephrin-B1^flox/y^ KO or tdTomatoERT2-CreGFAP control male mice, allowing for tdTomato expression in astrocytes and analysis of ephrin-B1 levels. All KO and control mice received tamoxifen at P14 intraperitoneally (0.5 mg in 5 mg/ml of 1:9 ethanol/sunflower seed oil solution) once a day for 5 consecutive days. Analysis was performed at P28 except for behavioral and PTZ seizure tests at P45–P50 (Fig. 1*A*).

10.1523/JNEUROSCI.0154-24.2024.t1-1 Table 1-1 Statistical analysis for figure 1. Download Table 1-1, DOCX file.

To achieve overexpression (OE) of ephrin-B1 in hippocampal astrocytes, we stereotaxically injected the adeno-associated viral particles (VPs) containing ephrin-B1 cDNA under GFAP promoter (AAV7.GfaABC1D.ephrin-B1.SV40; referenced in the text as AAV-ephrin-B1) into the hippocampus of P14 mice. Control AAV-tdTomato VP contained tdTomato cDNA under the same GFAP promoter (AAV7.GfaABC1D.tdTomato.SV40). AAV-ephrin-B1 VP (final concentration 7.56 × 1,012 VPs/ml) and control AAV-tdTomato VP (final concentration 4.46 × 1,012 VPs/ml) were both obtained from UPenn Vector Core (http://www.med.upenn.edu/gtp/vectorcore) and processed as previously described with modifications (Nguyen et al., 2020). VPs were concentrated with Amicon ultra-0.5 centrifugal filters (UFC505024, Sigma Millipore) pretreated with 0.1% Pluronic F-68 nonionic surfactant (24040032, Thermo Fisher Scientific).

We used several mouse lines to analyze the effects of ephrin-B1 OE in astrocytes as follows: (5) ChR2-YFP^flox/flox^ mice [Chr2-YFP:B6;129S-Gt(ROSA) 26Sortm32 (CAG-COP4pH134R/EYFP) Hze/J; RRID: IMSR_JAX#12569] were bred with mice expressing cre recombinase under the PV promotor [CrePV^+/+^; B6;129P2-Pvalbtm1(cre)Arbr/J; RRID: IMSR_JAX:017320] to obtain CrePV^+/−^ChR2-YFP^+/−^ mice expressing ChR2-YFP selectively in PV interneurons. This line was stereotaxically injected intrahippocampally with either AAV7.GfaABC1D.ephrin-B1.SV40 to overexpress astrocytic ephrin-B1 or AAV7.GfaABC1D.tdTomato.SV40 as a control and used for whole-cell recordings. (6) EphB2flox mice for conditional deletion of EphB2 using a Cre driver were generated by inserting the loxP site upstream of exon 2 and downstream of exon3. Deletion of this region should result in the loss of the ligand binding domain of EphB2 receptor. CrePV^+/+^ mice [B6;129P2-Pvalbtm1(cre)Arbr/J; RRID: IMSR_JAX:017320] were bred with EphB2^flox/flox^ mice to generate CrePV^+/−^EphB2^flox/+^ (het), CrePV^+/−^EphB2^flox/flox^ (KO), or littermate controls which either did not express Cre or did not contain the floxed EphB2. Mice from these groups were used for immunofluorescence staining analysis. Additionally, EphB2^flox/flox^ (KO) stereotaxically injected intrahippocampally with AAV7.GfaABC1D.ephrin-B1.SV40 to overexpress astrocytic ephrin-B1 and used for immunofluorescence staining analysis. (7) EphB2^flox/flox^ line was also bred with ChR2-YFP^flox/flox^ mice [Chr2-YFP:B6;129S-Gt(ROSA) 26Sortm32 (CAG-COP4pH134R/EYFP)Hze/J; RRID:IMSR_JAX#12569] to generate EphB2^flox/+^ChR2-YFP^flox/flox^ mice and was bred with CrePV^+/+^EphB2^flox/+^ mice to generate CrePV^+/−^EphB2^flox/flox^ ChR2-YFP^flox/+^ (KO) or CrePV^+/−^EphB2^+/+^-ChR2-YFP^flox/+^ (control) mice. This line expressed ChR2-YFP but lacked EphB2 in PV interneurons and was used for whole-cell electrophysiology recordings. Additionally, CrePV^+/−^EphB2^flox/flox^ChR2-YFP^flox/+^ (KO) mice were stereotaxically injected intrahippocampally with either AAV7.GfaABC1D.ephrin-B1.SV40 to overexpress astrocytic ephrin-B1 or AAV7.GfaABC1D.tdTomato.SV40 as a control and used for whole-cell recordings. Real-time PCR–based analysis of genomic DNA isolated from mouse tails was used to confirm genotypes for all mouse lines by Transnetyx. Analysis was performed at P28 except for behavioral and PTZ seizure tests at P45–P50 (Fig. 1*B*).

### Surgery

Mice were anesthetized with a ketamine/xylazine mixture (80 mg/kg ketamine and 10 mg/kg xylazine) given intraperitoneally. Paw pad pinch test, respiratory rhythm, righting reflex, and loss of corneal reflex were assessed to ensure the mice were adequately anesthetized. P14 B6/C57 mice (RRID:IMSR_JAX: 000664) received craniotomies (1 mm in diameter), and VPs were stereotaxically injected into the dorsal hippocampus (1.8 mm posterior to the bregma, 1.1 mm lateral to midline, and 1.3 mm from the pial surface; scaled to the appropriate coordinates based on bregma–lambda distance). Control mice were injected with 1 μl of 1.16 × 1,013 VP/ml AAV-tdTomato, and OE animals were injected with 1 μl of 3.78 × 1,013 VP/ml AAV-ephrin-B1. Prior to surgery, mice were injected subcutaneously with 3.25 mg/kg extended-release buprenorphine (Ethiqa XR) to manage pain. Additionally, mice received 5 mg/kg meloxicam every 12 h for pain management. Animals were allowed to recover for 14 d before analysis. P28 mice were used for immunohistochemistry, mRNA analysis, and whole-cell electrophysiology experiments. There was a significant increase in ephrin-B1 immunoreactivity in CA1 hippocampus on the injected ipsilateral side (OE) compared with noninjected contralateral control side (Fig. 1*F*,*G*).

### Immunohistochemistry

Immunohistochemistry was performed as described previously in Nguyen et al. (2020) with modifications. Animals were anesthetized with isoflurane and transcardially perfused with 0.1 M PBS followed by fixation with 4% paraformaldehyde (PFA) in 0.1 M PBS, pH 7.4. Brains were postfixed for 2 h or overnight at 4°C in either 2 or 4% PFA in 0.1 M PBS. One hundred micrometer coronal brain slices were obtained via vibratome sectioning. Samples were fixed for 30 min in 2% PFA, quenched with 50 mM NH4Cl, washed in 0.1% Tween in PBS (0.1% PBST), permeabilized in 0.1% Triton in PBS for 30 min, and blocked for 2 h in 10% normal donkey serum (NDS), 1% BSA, and 0.1% Triton in PBS. Primary and secondary antibodies were diluted in the buffer containing 1% NDS, 0.5% BSA, and 0.1% Triton in PBS. Primary antibodies were incubated overnight at 4°C. PV-positive cells and boutons were immunolabeled using mouse anti-PV antibody (1:500, Sigma-Aldrich, P3088, RRID:AB_477329). Inhibitory presynaptic sites were identified using rabbit anti-vesicular GABA transporter (VGAT) antibody (1:100, Synaptic Systems, 131002, RRID:AB_887871) and mouse anti-gephyrin antibody (1:500, Synaptic Systems, catalog #147111, RRID:AB_887719). EphB receptors 1-4 were detected using rabbit anti-EphB(1-4) antibody (1:200, Abcam, ab64820, RRID:AB_2278009). Ephrin-B1 was detected using goat anti-ephrin-B1 antibody (1:50, R&D Systems, AF473, RRID:AB_2293419). Astrocytes were identified using rabbit anti-GFAP antibody (1:500, Cell Signaling Technology, 12389, RRID:AB_2631098). EphB2 was labeled using goat anti-EphB2 antibody (1:50, Invitrogen, PA5-47017, RRID:AB_2609043). Following incubation with primary antibodies, samples were washed in 0.5% PBST and then incubated in secondary antibodies diluted in the same buffer for 2 h at RT. The following secondary antibodies were used: donkey anti-goat IgG 488 (1:500, Invitrogen, A-11055, RRID:AB_2534102), donkey anti-rabbit IgG 488 (1:500, Invitrogen, A-21206, RRID:AB_2535792), donkey anti-mouse IgG 488 (1:500, Invitrogen, A-21202, RRID:AB_141607), donkey anti-mouse IgG 594 (1:500, Invitrogen, A-21203, RRID:AB_141633), donkey anti-rabbit IgG 594 (1:500, Invitrogen, A-21207, RRID:AB_141637), donkey anti-rabbit IgG 647 (1:500, Invitrogen, A-31573, RRID:AB_2536183), and donkey anti-goat IgG 647 (1:500, Invitrogen, A-21447, RRID:AB_2535864). Samples were washed in 0.5% PBST, followed by PBS, and then mounted on coverslips with VECTASHIELD PLUS antifade mounting medium with DAPI (Vector Laboratories, H-2000). Astrocytes were also visualized with tdTomato in ephrin-B1 KO mice (Fig. 1*C*–*E*), and astrocyte immunoreactivity was assessed with GFAP in ephrin-B1 OE mice (Fig. 1*F–H*).

### Confocal imaging and analysis

Confocal images of coronal brain slices containing the stratum oriens (SO), stratum pyramidale (SP), and stratum radiatum of the dorsal CA1 hippocampus were taken using Zeiss LSM 880 inverted laser scanning microscope and Zeiss LSM 880 upright laser scanning microscope. High-resolution optical sections were acquired with a 20× air objective (0.8 NA), 1× zoom at 1 μm step intervals to measure PV, EphB, and Ephrin-B1 immunoreactivity. Confocal images of synaptic puncta, including PV, VGAT, and gephyrin, and high-magnification images of Ephrin-B1/EphB receptors and GFP-labeled soma and dendrites were taken using an air 40× (1.2 NA) or oil 63× objectives, (1.4 NA), at 1× zoom (1,024 × 1,024) or (2,048 × 2,048) pixel format, with a 0.5 μm step size. Images were acquired and processed under identical conditions for analysis.

For analysis of VGAT/PV colocalization, images were analyzed using the Neurolucida 360 (MBF Bioscience, RRID:SCR_016788) and Neurolucida Explorer software (MBF Bioscience, RRID:SCR_017348). *Z*-stacks were imported into Neurolucida, background subtracted, and histogram equalized, and contrast was enhanced. Image stacks were trimmed in the *Z* plane to analyze stacks of an equal number of *Z* planes, and contours were drawn around regions of interest (ROIs) to be analyzed. 3D renderings were generated by rendering all VGAT puncta >0.15 μm^3^ within the ROI, followed by rendering PV puncta that were at least 0.15–0.2 μm^3^ in size and were at most 0.5–1.0 μm from a VGAT puncta. If the images contained background VGAT somatic staining, puncta rendered in the soma were removed prior to rendering PV boutons. Images were analyzed in Neurolucida 360 explorer by selecting puncta within the ROI and measuring the total number of puncta as well as the number of colocalized puncta showing at least 10% colocalization using the puncta feature. The number of colocalized puncta was normalized to the 3D volume.

For analysis of VGAT/PV and VGAT/gephyrin colocalization in close proximity to Thy1-GFP or tdTomato-expressing excitatory cells in Neurolucida 360, images were processed as described above, excitatory cells were rendered using soma and tree tracing, and then VGAT and PV boutons or gephyrin puncta within 1 μm of the cell that were at least 0.15 μm^3^ in size were rendered using the puncta feature. Renderings were analyzed in Neurolucida 360 by selecting the soma or dendrites and analyzing the number of colocalized puncta showing at least 10% colocalization near the selected soma or dendrite. The number of colocalized VGAT/PV and VGAT/gephyrin puncta was normalized to the volume of the selected soma or dendrite.

Analysis of EphB/PV puncta colocalization in the SP was performed in Neurolucida 360 software. *Z*-stacks were imported into Neurolucida 360, background subtracted, and histogram equalized, and contrast was enhanced. Image stacks were trimmed in the *Z* plane to analyze stacks of an equal number of *Z* planes, and contours were drawn around ROIs to be analyzed. 3D renderings of PV boutons of at least 0.15 μm^3^ and EphB puncta of at least 0.05 μm^3^ were generated in Neurolucida 360, and then renderings were analyzed in Neurolucida 360 by selecting puncta within the ROI, measuring the total number of puncta and the number of colocalized puncta showing at least 10% colocalization using the puncta feature, then normalizing to the 3D volume.

Analysis of EphB puncta on PV soma was performed in ImageJ. *Z*-stacks of equal size were collapsed into a single image by projection, converted to a tiff file, and background subtracted. Each image was threshold-adjusted and converted into a binary image, the despeckle function was used to eliminate noise, and the watershed function was used to segment overlapping puncta. ROIs were drawn around PV soma and the number of puncta per soma was counted using the particle analysis tool, where puncta of at least 0.1 μm^2^ in size were analyzed. The number of puncta was normalized to the area of each soma.

Analysis of EphB/Ephrin-B1 colocalized puncta in SP and on PV soma was performed in ImageJ in a similar way as described above. *Z*-stacks of equal size were collapsed into single image by *z*-projection, converted to a tiff file, and background subtracted. Each image was split into individual channels, threshold-adjusted, and converted into a binary image, the despeckle function was used to eliminate noise, and the watershed function was used to segment overlapping puncta. ROIs were drawn around the SP and PV soma, and the number of colocalized puncta per soma was counted using the particle analysis tool, where puncta of 0.1–10 μm^2^ in size were analyzed. The number of puncta was normalized to the area of each soma.

Analysis of EphB2 and PV immunofluorescence levels in PV interneurons was performed in the ImageJ software. For EphB2 analysis, single image planes were separated by channel, ROIs were drawn around PV somata, and then EphB2 mean fluorescence intensity levels within the ROIs were measured. The background signal was measured using a secondary antibody only control and then was subtracted from the fluorescence intensity measurement. For PV analysis, *Z*-stacks of equal size were collapsed into a single image by projection and converted to a tiff file. ROIs were drawn around PV soma, and then PV mean fluorescence intensity levels within the ROIs were measured.

### Electron microscopy

P28 EphB2 KO and control mice were perfused first with Ringers containing (in mM) 125 NaCl, 2 CaCl_2_, 5 KCl, and 0.8 NaH_2_PO_4_, pH adjusted to 7.4, then with fixative containing 2.5% glutaraldehyde (EMS, 111-30-8), 2% PFA (EMS, 15710) in 0.15 M cacodylate buffer (111-30-8). Brains were postfixed overnight in the same fixative solution. The 200-μm-thick vibratome sections were sliced in prechilled 0.15 M cacodylate buffer (111-30-8), and then slices were immediately returned to the fixative solution. Perfusion-fixed tissues were postfixed in 1% OsO4 in 0.1 M cacodylate buffer for 1 h on ice and then stained with 2% uranyl acetate for 1 h on ice. The tissues were dehydrated in a graded series of ethanol washes (50–100%) once, followed by a wash with 100% ethanol and two washes with acetone for 15 min each, and embedded with Durcupan. Seventy nanometer sections were cut on a Leica UCT ultramicrotome and collected on 300 mesh copper grids. Sections were stained with 2% uranyl acetate for 5 min and Sato lead stain for 1 min. Samples were viewed using a JEOL 1400-plus TEM (JEOL). Transmission electron microscopy (EM) images were taken using a Gatan OneView 4 × 4 k camera at 4,000× and 8,000× magnification (Gatan).

Analysis of perisomatic presynaptic sites and glial processes near neuronal soma was performed using 4,000× images in Adobe Photoshop. ROIs were drawn using lasso function around perisomatic presynaptic boutons or glial processes to measure their size. Perisomatic boutons were identified by the presence of large presynaptic vesicles and a close proximity to neuronal soma. Active zones were detected by the presence of symmetric synaptic junction with the adjacent cluster of synaptic vesicles. Glial processes were identified around presynaptic boutons as structures with pale cytoplasm and undefined shapes.

### Whole-cell patch–clamp recordings

Animals were anesthetized with isoflurane, brains were dissected, and 300 μm coronal sections were sliced in ice-cold sucrose containing ACSF containing (in mM) 2.5 CaCl2, 196.6 sucrose, 2.5 KCl, 1.3 MgSO4, 1 NaH2PO4, 26.2 NaHCO3, 11 d-glucose, 3 kyneuric acid, 2 Na-pyruvate, and 3.5 MOPS. Slices were incubated in ice-cold sucrose ACSF for 30 min. Slices were then transferred to ACSF containing (in mM) 126 NaCl, 2.5 KCl, 10 d-glucose, 1.25 NaH2PO4, 2 MgCl2, 2 CaCl2, and 26 NaHCO3 and allowed to incubate for 45 min at room temperature. Slices were transferred to a recording chamber and continuously perfused with oxygenated ACSF at a flow rate of 1 ml/min at 31°C and allowed to equilibrate to the chamber for at least 10 min.

Whole-cell patch experiments were conducted blind, with experimental procedures described in Castañeda-Castellanos et al. (2006). Blind, whole-cell patch–clamp recordings were obtained from CA1 pyramidal neurons using pipettes made from glass capillaries pulled by Narishige PC-10 vertical micropipette puller (Narishige, RRID:SCR_022057). Pipette resistance ranged from 3 to 5 mOhms and were filled with high-chloride internal solution containing (in mM) 125 KCl, 10 K-gluconate, 2 MgCl2, 0.2 EGTA, 10 HEPES, 2 Mg2ATP, 0.5 Na2GTP, 10 PO creatine, and 10 QX-314 and 0.2% biocytin for postlabeling and was pH adjusted to 7.2 with KOH. CA1 hippocampal neurons were voltage clamped and held at −60 mV. Due to high-chloride internal solution and negative holding potential, inhibitory currents were measured as inward currents. PCs were voltage clamped at −60 mV, and IPSCs were recorded in response to photoactivation.

PV interneurons were optically stimulated using a fiber-coupled LED light of 470 nm wavelength (Thorlabs, LEDD1B). A cannula was attached to the fiber-optic cable in order to submerge the light source into the bath and place the light source directly over the slice. Input–output curves were generated using a fixed duration of light pulse (5 ms) and increasing the intensity of the LED power. Twelve sweeps with increasing intensity of the LED light source were used (starting at ∼0.13 mW and increasing up to 4 mW of power) to photoactivate PV interneurons and to record IPSCs in PCs. A 20 Hz stimulation was also used in order to analyze short-term plasticity, where oeIPSC amplitudes were measured from the original baseline prior to stimulation to the peak of each oeIPSC in the train. We used a 5 ms LED pulse followed by 45 ms of LED off at 100% LED power in this protocol. oeIPSCs were recorded using a HEKA EPC-9 amplifier (HEKA Elektronik), filtered at 1 kHz, and digitized at 10 kHz. Series resistance was compensated for and was monitored throughout the experiment by delivering 5 mV voltage steps. Data were discarded if the series resistance changed >20% during the course of an experiment. Data were analyzed on a HEKA Elektronik software, and statistical significance was analyzed using the Prism 10 software (GraphPad Software, RRID:SCR_002798).

### PTZ seizure test

Before testing, mice were housed in a room with a 12 h light/dark cycle with *ad libitum* access to food and water. The cages were transferred to the behavioral room for 30 min habituation prior to the test. Between tests, the cages were cleaned with 2–3% acetic acid, 70% ethanol, and water to eliminate odor trails. Pentylenetetrazole (PTZ) (Sigma-Aldrich, P6500) was diluted in 0.9% sodium chloride injection diluent (Hospira, NDC-0409-4888-03) to make a stock solution of 5 mg/ml. Ephrin-B1 KO and their controls received three doses of PTZ, including 20, 30, and 40 mg/kg, based on the weight of the mouse, over the 5 h testing period that were delivered via intraperitoneal injection. At time *t* = 0 h, mice received a 20 mg/kg dose of PTZ and were recorded for 1 h. At *t* = 2 h, mice received a 30 mg/kg dose of PTZ and were recorded for 1 h. At *t* = 4 h, mice received a 40 mg/kg dose of PTZ and were recorded for 1 h. EphB2 KO and their controls received 40 and 50 mg/kg doses of PTZ via intraperitoneal injection over the course of the 1 h testing period. At time *t* = 0 h, mice received a 40 mg/kg dose of PTZ and were recorded for 30 min. At time *t* = 0.5 h, mice received a 50 mg/kg dose of PTZ and were recorded for another 30 min. Data were analyzed based on the Racine seizure score as described in Shimada and Yamagata (2018). Videos were manually scored by an experimenter blind to the condition as follows: Score 0, the absence of seizure activity; Score 1, immobilization, lying flat on the abdomen; Score 2, head nodding, facial, forelimb, or hindlimb myoclonus; Score 3, continuous whole-body myoclonus, myoclonic jerks, tail held up stiffly; Score 4, tonic–clonic seizure, rearing, and falling; Score 5, tonic–clonic seizure, loss of postural tone, wild rushing, and jumping; and Score 6, death.

### Behavior

Home cage behaviors were analyzed as described by Reinhard et al. (2019) with modifications. Mice were placed in new cages with fresh bedding and recorded for 30 min. Videos were manually scored by an experimenter blind to the condition. The video was paused at 10 s intervals for the first 10 min of the recording, and the observer recorded if the mouse was performing any of the following: motion, rearing, digging, grooming, or scanning.

### Open-field test

Anxiety-like behaviors and locomotor activity were tested in experimental mice as described previously with modifications (Dansie et al., 2013; Lovelace et al., 2020; Rais et al., 2022). The cages with mice were transferred to the behavioral room 30 min before the testing. A 72 × 72 cm open-field (OF) arena with 50-cm-high walls was constructed from opaque acrylic sheets with a clear acrylic sheet for the bottom. The OF arena was placed in a brightly lit room, and one mouse at a time was placed in a corner of the OF and allowed to explore for 10 min while being recorded with digital video from above. The floor was cleaned with 2–3% acetic acid, 70% ethanol, and water between tests to eliminate odor trails. The mice were tested between the hours of 8:00 A.M. and 2:00 P.M. The arena was subdivided into a 4 × 4 grid of squares with the middle of the grid defined as the center. A line 4 cm from each wall was added to measure thigmotaxis. Locomotor activity was scored by the analysis of total line crosses and speed using the TopScan Lite software (Clever Sys). The analysis was performed for the first and second 5 min of the testing. Assessments of the digital recordings were performed blind to the condition.

### Social novelty test

Sociability and social memory were studied using a three-chamber test as described previously with minor modifications (Kaidanovich-Beilin et al., 2011; Nguyen et al., 2020). Briefly, a rectangular box contained three adjacent chambers 19 × 45 cm each, with 30-cm-high walls and a bottom constructed from clear Plexiglas. The three chambers were separated by dividing walls, which were made from clear Plexiglas with openings between the middle chamber and each side chamber. Removable doors over these openings permitted chamber isolation or *ad libitum* access to all chambers. All testing was done in a brightly lit room (650 lux), between 9:00 A.M. and 4:00 P.M. The test mouse was placed in the central chamber with no access to the left and right chambers and allowed to habituate to the test chamber for 5 min before testing began. Session 1 measured sociability. In Session 1, another mouse (Stranger 1) was placed in a wire cup-like container in one of the side chambers. The opposite side had an empty cup of the same design. The doors between the chambers were removed, and the test mouse was allowed to explore all three chambers *ad libitum* for 10 min while being digitally recorded from above. The following parameters were monitored: the duration of direct contact between the test mouse and either the stranger mouse or empty cup and the duration of time spent in each chamber. Session 2 measured social memory and social novelty preference (SNP). In Session 2, a new mouse (Stranger 2) was placed in the empty wire cup in the second side chamber. Stranger 1, a now familiar mouse, remained in the first side chamber. The test mouse was allowed to freely explore all three chambers for another 10 min while being recorded, and the same parameters were monitored. Placement of Stranger 1 in the left or right side of the chamber was randomly altered between trials. The floor of the chamber was cleaned with 2–3% acetic acid, 70% ethanol, and water between tests to eliminate odor trails. Assessments of the digital recordings were done using the TopScan Lite software (Clever Sys). To measure changes in sociability and social memory, we calculated the percentage time spent in each chamber in each test. Furthermore, a $\mathrm{sociability}\;\mathrm{index}\;=\left(\frac{\mathrm{time}\;\mathrm{in}\;S1\;\mathrm{chamber}}{\mathrm{time}\;\mathrm{in}\;S1\;\mathrm{chamber}\;+\;\mathrm{time}\;\mathrm{in}\;\mathrm{empty}\;\mathrm{chamber}}\right)$ and $\mathrm{social}\;\mathrm{novelty}\;\mathrm{preference}\;\mathrm{index}=\left(\frac{\mathrm{time}\;\mathrm{in}\;S2\;\mathrm{chamber}}{\mathrm{time}\;\mathrm{in}\;S2\;\mathrm{chamber}\;+\;\mathrm{time}\;\mathrm{in}\;S1\;\mathrm{chamber}}\right)$ were calculated as described previously (Nguyen et al., 2020). For sociability index, values <0.5 indicate more time spent in the empty chamber, >0.5 indicate more time spent in the chamber containing Stranger 1, and 0.5 indicates equal amount of time in both chambers. For SNP index, values <0.5 indicate more time spent in the chamber containing Stranger 1 or now familiar mouse, >0.5 indicate more time spent in the chamber containing Stranger 2 or new stranger mouse, and 0.5 indicates equal amount of time in both chambers.

### Marble burying test

Compulsive–obsessive-like behaviors were evaluated using the marble burying test as described in Deacon (2006) and Angoa-Pérez et al. (2013) with minor modifications. Briefly, standard polycarbonate mouse cages with fitted filter-top covers were used for the marble burying test. Fresh unscented mouse bedding material was added to each cage to a depth of 4 cm and further leveled. Standard glass toy marbles (assorted styles and colors, 15 mm diameter, 5.2 g in weight) were gently placed on the surface of the bedding in four rows of five marbles. The marbles were washed in warm laboratory detergent, rinsed thoroughly in distilled water and dried prior to each experiment. One mouse was placed into a corner of the cage containing marbles, being careful not to disturb the marbles, and the filter-top cover was placed on the cage. Food and water were withheld during the testing. A mouse was allowed to remain in the cage undisturbed for 30 min. After the test completion, the mouse was returned to its home cage, 20 marbles were retrieved, and the bedding was disposed. Pictures of the cage with marbles were taken before the test and after the test completion. The number of buried and displaced marbles was counted. Marbles were scored as buried if two-thirds of their surface area was covered by bedding.

### Statistical analyses

#### Immunohistochemistry analysis

For VGAT/PV colocalization in the SP, statistical analysis was performed using the GraphPad Prism 10 software (RRID:SCR_002798). Astrocytic ephrin-B1 OE mice were analyzed by two-tailed *t* tests, five male mice with 10 images per group. Astrocytic ephrin-B1 KO and CON mice were analyzed using a two-tailed *t* test, three KO and three CON male mice per group with six images per group. PV-EphB2 control, heterozygous, and KO male and female mice were analyzed by Brown–Forsythe and Welch ANOVA test in the GraphPad Prism 10 software (RRID:SCR_002798), *n* = 5–13 mice per group and 19–46 images per group. PV-EphB2 KO + AAV-EfnB1 male and female mice were analyzed by two-tailed *t* tests, *n* = 22 images/group, six mice. For analysis of VGAT/PV near Thy1-GFP excitatory neurons, statistical analysis was performed using the GraphPad Prism 10 software (RRID:SCR_002798) using Welch-corrected two–tailed *t* tests, four male mice per group and 13–18 images per group. For analysis of VGAT/gephyrin colocalization on SP somata, statistical analysis was performed using the GraphPad Prism 10 software (RRID:SCR_002798) using Welch-corrected two–tailed *t* test, four mice per group and 30–31 cells per group. For analysis of EphB puncta and PV/EphB puncta in the SP, statistical analysis was performed using the GraphPad Prism 10 software (RRID:SCR_002798) using two-tailed *t* test, 3–4 male mice per group and 8–16 images per group. For analysis of EphB/ephrin-B1 colocalization in SP, statistical analysis was performed using the GraphPad Prism 10 software (RRID:SCR_002798) using Welch-corrected *t* tests, 3–4 mice per group (KO) or 5 mice per group (OE). For analysis of EphB puncta on PV somata and EphB/ephrin-B1 colocalization on PV somata, statistical analysis was performed using the GraphPad Prism 10 software (RRID:SCR_002798) using two-tailed *t* test, 3–4 male mice per group and 10–25 PV interneurons per group. PV and EphB2 immunoreactivity in PV interneurons, statistical analysis was performed using the GraphPad Prism 10 software (RRID:SCR_002798). Two-tailed *t* test was used for analysis of PV immunoreactivity in PV interneurons of astrocytic ephrin-B1 OE (3 OE male mice per group with 19–27 cells per group) and KO mice (4 male mice per group with 23–24 cells per group). Brown–Forsythe and Welch ANOVA with Dunnett's multiple-comparisons test were used for analysis of PV immunoreactivity in PV interneurons of PV-EphB2 control, heterozygous, or KO male and female mice (72–165 cells per group, 4–13 mice per group). Two-tailed *t* test was used to analyze PV interneuron density in OE and EphB2 KO groups, 4 mice per group (OE) or 5 mice per group (EphB2 KO). Welch's *t* test was used for analysis of EphB2 levels in PV interneurons of PV-EphB2 KO and control male and female mice (*n* = 3–4 mice per group, 50–57 cells per group).

For EM analysis, statistical analysis was performed using the GraphPad Prism 10 software. Two-tailed *t* test or two-way ANOVA (presynaptic size distribution), Sidak multiple-comparison test were used to compare controls and EphB2 KO mice, three mice per group.

#### Electrophysiology analysis

Unpaired Student's two-tailed *t* test was used to analyze peak oeIPSC amplitude, and two-way ANOVA with Sidak's multiple-comparison post hoc test was used to analyze oeIPSC amplitude against LED power, oeIPSCs over 20 Hz trains, and paired pulse ratios (PPRs). AAV-EfnB1/AAV-TdTomato male and female mice were with 8–11 cells per group and 7–8 mice per group. PV-EphB2 KO/CON male and female mice were with 18 cells per group and 7–9 mice per group. PV-EphB2 KO+AAV-EfnB1/PV-EphB2 KO+AAV-TdTomato male and female mice were with 9–12 cells per group 5–6 mice per group.

#### Behavior analysis

For PTZ seizures, statistical analysis was performed using the GraphPad Prism 10 software (RRID:SCR_002798). Latency to tonic–clonic seizure was analyzed by two-tailed *t* test, seizure duration was analyzed with two-tailed *t* test or Mann–Whitney test, and the number of seizure events or mice showed seizures were analyzed with two-tailed *t* test, Mann–Whitney test, or odds ratio, 8–12 male mice per group.

For home cage behaviors, statistical analysis was performed using the GraphPad Prism 10 software (RRID:SCR_002798) using Mann–Whitney tests, 12 male mice per group. For marble burying test, statistical analysis was performed using the GraphPad Prism 10 software (RRID:SCR_002798) using unpaired *t* tests, 9–16 mice per group. For three-chamber sociability and social novelty test, statistical analysis was performed using the GraphPad Prism 10 software (RRID:SCR_002798) using unpaired *t* tests, 11–16 mice per group. For the OF test, statistical analysis was performed using the GraphPad Prism 10 software (RRID:SCR_002798) using two-way ANOVA with Fisher's exact LSD (time in OF) or *t* test (distance traveled), 9–16 mice per group.

## Results

### Astrocytic ephrin-B1 promotes functional connectivity between PV and PCs

To test if astrocytes regulate the development of functional inhibitory connections between PV interneurons and pyramidal neurons via EphB receptor signaling, we manipulated the levels of the EphB receptor ligand, ephrin-B1, in astrocytes during the early P14–P28 developmental period (Fig. 1*A,B*). We administered tamoxifen to tdTomatoERT2-*Cre^GFAP^ephrin-B1*^flox/y^ KO or tdTomatoERT2-Cre*^GFAP^* control mice and analyzed the levels of astrocytic ephrin-B1 and astrocyte density (Fig. 1*C–E*). Immunofluorescence intensity of ephrin-B1 in tdTomato-expressing astrocytes was significantly reduced in KO mice (Fig. 1*D*; Extended Data; *t* test; *t*_(18)_ = 4.280; *p* = 0.0005); however the density of astrocytes in the CA1 hippocampus was unaffected by astrocytic ephrin-B1 deletion (Fig. 1*E*; Extended Data; *t* test; SP, *t*_(121)_ = 0.8369; *p* = 0.4043; all layers, *t*_(121)_ = 0.7623; *p* = 0.4474). We overexpressed ephrin-B1 in hippocampal astrocytes of P14 male and female mice by injecting AAV7.GfaABC1D.ephrin-B1.SV40 (AAV-EfnB1) into the hippocampus, while vehicle-injected controls received AAV7.GfaABC1D.tdTomato.SV40 (AAV-TdTomato; Fig. 1*B*,*F*). We observed a significant upregulation of ephrin-B1 in GFAP-positive astrocytes following the injection with AAV-EfnB1 (Fig. 1*G*; Extended Data; *t* test; *t*_(10)_ = 3.629; *p* = 0.0046), but no effects of viral injections on GFAP immunoreactivity in CA1 hippocampus compared with the contralateral noninjected side (Fig. 1*H*; Extended Data; *t* test; SP, *t*_(27)_ = 1.412; *p* = 0.1695; SO, *t*_(27)_ = 0.4519; *p* = 0.6549). PV→PC connectivity was tested at P28+/−2D using whole-cell voltage–clamp electrophysiology in combination with an optogenetic approach to selectively activate PV-expressing interneurons. PV interneurons expressing ChR2-YFP were optogenetically activated using 470 nm LED light, and optically evoked, gabazine-sensitive IPSCs (oeIPSCs) were recorded in CA1 PCs of astrocytic ephrin-B1 OE mice and vehicle-injected control mice (Fig. 1*I*,*J*). Input–output (IO) curves were generated by delivering LED light pulses with increasing LED power at a fixed pulse length, and the maximum responses were analyzed to measure peak oeIPSC amplitude (Fig. 1*J*–*L*). We observed a significant effect of LED power and interaction of genotype and LED power (Fig. 1*K*; Extended Data; two-way ANOVA; Sidak's multiple-comparison test; *F*_(11,231)_ = 2.684; *p* = 0.0029). In addition, OE of ephrin-B1 in astrocytes significantly increased the peak oeIPSC amplitude, suggesting that astrocytic ephrin-B1 positively regulates PV→PC functional connectivity (Fig. 1*L*; Extended Data; *t* test; *t*_(21)_ = 2.303; *p* = 0.0316). To test if astrocytic ephrin-B1 also affects short-term plasticity at PV→PC synapses, we optically simulated PV interneurons with a 20 Hz train of 10 LED light pulses at maximal LED power (Fig. 1*M*). We observed a significant increase in the average oeIPSC amplitude during the first two stimulations in the OE group, consistent with the increased strength of the functional connections between PV interneurons and CA1 PCs following the OE of astrocytic ephrin-B1 (Fig. 1*N*; Extended Data; two-way ANOVA; *F*_(1,17)_ = 4.221; *p* = 0.0556; Sidak's multiple-comparisons post hoc test; *p* < 0.0001; *p* = 0.0237). However, the increase in oeIPSC amplitude was not accompanied by any significant difference in the PPR of the oeIPSCs, calculated as a ratio of the oeIPSC amplitude for each pulse to the first oeIPSC amplitude (Fig. 1*O*; Extended Data; two-way ANOVA; Sidak's multiple-comparison post hoc test; *F*_(1,17)_ = 0.3607; *p* = 0.5560), suggesting no differences in release probability. Our data suggest that the observed increase in the strength of PV→PC connectivity following OE of ephrin-B1 in astrocytes is most likely a result of increased PV→PC synapse number or their strength.

### Astrocytic ephrin-B1 boosts the development of PV-specific presynaptic inhibitory sites

To test if levels of astrocytic ephrin-B1 affect the development of structural PV-positive inhibitory synapses during the P14–P28 period, we analyzed the density of PV-positive inhibitory presynaptic sites in the pyramidal (SP) layer of the CA1 hippocampus with immunostaining. Astrocytic ephrin-B1 was overexpressed in hippocampal astrocytes at P14, and analysis was done at P28 with the contralateral, noninjected side used as a control (Fig. 1*B*). PV-positive presynaptic sites were detected with antibodies against PV and VGAT in brain slices (Fig. 1*P*,*Q*). OE of astrocytic ephrin-B1 increased the number of VGAT/PV colocalized puncta in the SP (Fig. 1*R*; Extended Data; *t* test; *t*_(18)_ = 2.322; *p* = 0.0322). Interestingly, PV levels (Fig. 1*S*; Extended Data; *t* test; *t*_(44)_ = 1.616; *p* = 0.1132) and the density of PV-expressing cells (Fig. 1*T*; Extended Data; *t* test; *t*_(32)_ = 1.460; *p* = 0.1541) were not significantly different between OE and control groups. The data suggest that astrocytic ephrin-B1 positively regulates the establishment of PV-positive presynaptic sites in the CA1 hippocampus during development.

### Inhibitory innervation of CA1 pyramidal neurons by PV interneurons is reduced following developmental deletion of astrocytic ephrin-B1

**Figure 2.**
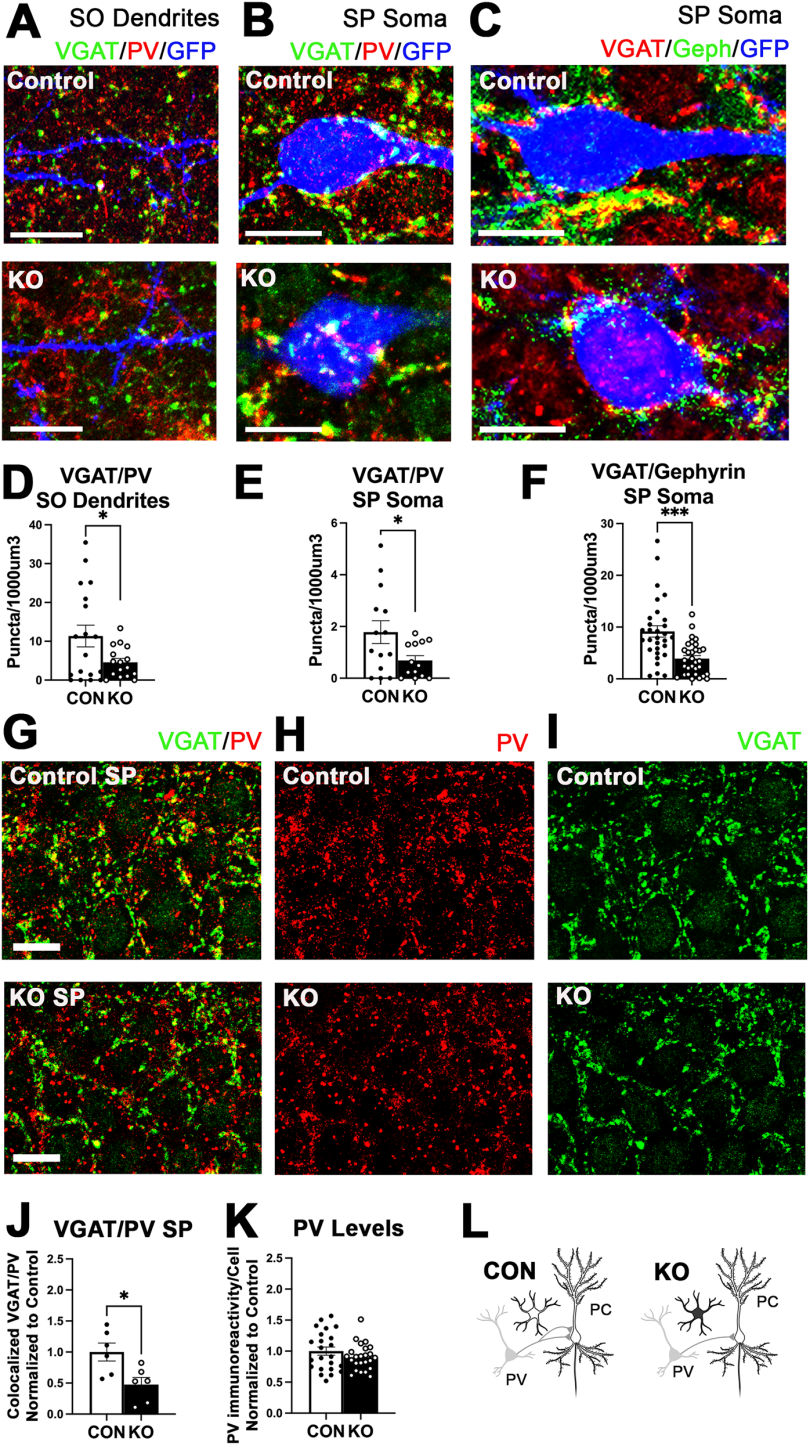
Deletion of astrocytic ephrin-B1 reduces PV perisomatic innervation of CA1 pyramidal neurons. ***A***, ***B***, Confocal images showing VGAT (green) and PV (red) immunostaining on SO dendrites (***A***) or somata (***B***) of CA1 excitatory pyramidal neurons labeled with GFP (blue) in control and KO mice. Scale bar, 10 μm. ***C***, Confocal images showing VGAT (red) and gephyrin (green) immunostaining on somata of CA1 excitatory pyramidal neurons labeled with GFP (blue) in control and KO mice. Scale bar, 10 μm. ***D***, ***E***, Graphs represent the average colocalized VGAT/PV puncta on SO dendrites (***D***) or somata (***E***) of CA1 excitatory neurons in control and astrocytic ephrin-B1 KO mice. KO mice show a significantly reduced density of colocalized VGAT/PV puncta on SO dendrites and somata of excitatory neurons (13–18 images/4 mice per group; *t* test; *p < 0.05; Extended Data Table 2-1). ***F***, The graph shows the average number of colocalized VGAT/gephyrin-positive puncta on CA1 excitatory neuronal soma. KO mice show a significant reduction in the density of VGAT-/gephyrin-positive puncta (30–31 cells/4 mice per group; Welch-corrected *t* test; ***p < 0.0001; Extended Data Table 2-1). ***G–I***, Confocal images of SP layer of CA1 hippocampus immunolabeled against VGAT (green) and PV (red); scale bar, 25 μm. ***J***, The graph shows the average number of colocalized VGAT/PV puncta in the KO group normalized to the control. The KO group shows a reduction in VGAT/PV colocalized puncta in the SP layer of the CA1 hippocampus (6 images/3 mice per group; *t* test; *p < 0.05; Extended Data Table 2-1). ***K***, The graph shows the PV immunoreactivity levels within PV interneurons of KO and control mice (***N***). PV levels in PV interneurons were not affected by KO (23–24 cells/4 mice per group, *t* test, Extended Data Table 2-1). ***L***, The drawing depicts the changes in PV-positive innervation of PC following deletion of astrocytic ephrin-B1. All data are represented as mean ± SEM. Graphics created with Biorender.com.

To examine if somatic innervation of CA1 PCs was specifically disrupted by the deletion of ephrin-B1 in astrocytes, we analyzed the innervation of soma and dendrites of CA1 PCs by PV cells. PV-positive presynaptic sites onto GFP-expressing CA1 pyramidal neurons were detected using immunostaining against VGAT and PV. We observed a significant reduction in the number of colocalized VGAT/PV presynaptic sites in the SP of KO mice (Fig. 2*J*; Extended Data; *t* test; *t*_(10)_ = 2.841; *p* = 0.0175) and more specifically on both the SO dendrites (Fig. 2*D*; Extended Data; Welch-corrected *t* test; *t*_(21.21)_ = 2.277; *p* = 0.0332) and somata (Fig. 2*E*; Extended Data; Welch-corrected *t* test; *t*_(17.37)_ = 2.290; *p* = 0.0348) of CA1 pyramidal neurons in the hippocampus of P28 ephrin-B1 KO mice compared with controls. Additionally, there was a significant reduction in the number of colocalized VGAT-/gephyrin-positive puncta on the soma of CA1 PCs (Fig. 2*F*; Extended Data; Welch-corrected *t* test; *t*_(43.61)_ = 4.213; *p* = 0.0001), suggesting that the reduced number of VGAT/PV puncta was due to a reduced number of synapses and not due to our inability to detect PV. Indeed, PV levels were not significantly different in the KO group compared with controls (Fig. 2*K*; Extended Data; *t* test; *t*_(45)_ = 1.247; *p* = 0.2187). The data indicate that developmental deletion of astrocytic ephrin-B1 reduces the number of PV-positive structural synapses formed onto both SO dendrites and somata of CA1 excitatory neurons and suggests that PV→PC connectivity is regulated by the levels of astrocytic ephrin-B1.

10.1523/JNEUROSCI.0154-24.2024.t2-1 Table 2-1 Statistical analysis for figure 2. Download Table 2-1, DOCX file.

### Deletion of astrocytic ephrin-B1 increases seizure susceptibility and repetitive behaviors

**Figure 3.**
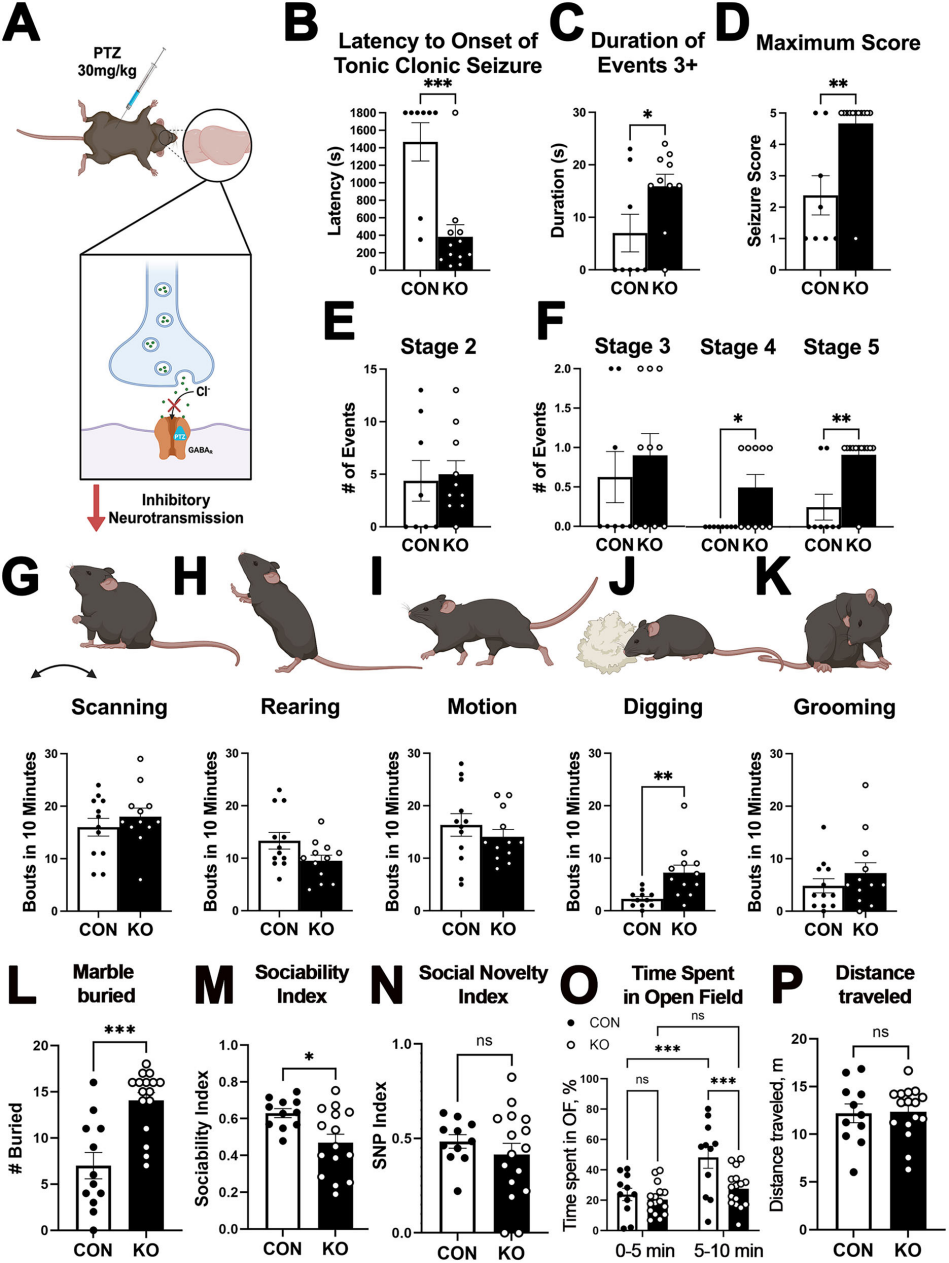
Deletion of astrocytic ephrin-B1 leads to increased seizure susceptibility, anxious and repetitive behaviors, as well as reduced sociability. ***A***, The diagram shows seizure testing paradigm and the mechanism of action of PTZ. ***B***, The graph shows the latency to the onset of tonic–clonic seizure in control and KO mice following injection of 30 mg/kg PTZ at P48+/−2D. Astrocytic ephrin-B1 deletion reduced the latency to the onset of tonic–clonic seizure following PTZ treatment, indicating that KO mice were more susceptible to PTZ-induced seizure (8–12 mice/group; *t* test; ***p < 0.001; Extended Data Table 3-1). ***C***, ***D***, The duration of events stage 3+ (***C***) and maximum seizure score achieved (***D***) as measured by racine seizure scale were increased following deletion of astrocytic ephrin-B1, indicating that deletion of astrocytic ephrin-B1 increased the severity of PTZ-induced seizures (8–12 mice/group; *t* test or Mann–Whitney test; *p < 0.05; **p < 0.01; Extended Data Table 3-1). ***E***, ***F***, Graphs show the number of seizure events of Stages 2–5 as measured by the Racine seizure scale. KO mice show increased numbers of events at Stages 4 and 5 compared with control mice (8–12 mice/group; Mann–Whitney test; *p < 0.05; **p < 0.01; Extended Data Table 3-1). ***G–K***, Graphs show analysis of common home cage behaviors represented as the number of scanning, grooming, motion, digging, and grooming bouts in 10 min. KO mice displayed an increased number of digging bouts compared with control mice at P28 **(*J***; 12 animals per group; *t* test; **p < 0.01; Extended Data Table 3-1). ***L–P***, Graphs show the analysis of marble burying (***L***), social behaviors in three-chamber test (***M***, ***N***), and anxiety-like behaviors in OF test (***O***, ***P***; 11–16 mice/group; *t* test or two-way ANOVA; *p < 0.05; ***p < 0.001; Extended Data Table 3-1). Ephrin-B1 KO mice showed increased obsessive–compulsive behaviors, reduced sociability, and anxiety-like behaviors. All data are represented as mean ± SEM. Graphics created with Biorender.com.

To assess whether alterations in PV-mediated inhibition of CA1 pyramidal neurons led to functional changes in astrocytic ephrin-B1 KO mice, we examined their susceptibility to seizures induced by PTZ, a GABA_A_R antagonist (Fig. 3*A*). Indeed, P45–P50 mice lacking astrocytic ephrin-B1 were more susceptible to PTZ-induced seizures and exhibited robust seizures in response to PTZ at a 30 mg/kg dose, while only 25% of the controls seized during the duration of the test. The latency to seizure was also significantly reduced in KO mice (Fig. 3*B*; Extended Data; *t* test; *t*_(18)_ = 4.439; *p* = 0.0003), while the duration (Fig. 3*C*; Extended Data; *t* test; *t*_(16)_ = 2.166; *p* = 0.0458) and maximum seizure score (Fig. 3*D*; Extended Data; Mann–Whitney test; DoF(18); MWU = 17; *p* = 0.0044) were significantly increased compared with controls. The increased maximum score was due to an increase in the number of Stage 4 (Fig. 3*F*; Extended Data; Mann–Whitney test; DoF(16); MWU = 20; *p* = 0.0359) and Stage 5 events in KO mice compared with controls (Fig. 3*F*; Extended Data; Mann–Whitney test; DoF(18); MWU = 16; *p* = 0.0044). In addition, we found that astrocytic ephrin-B1 KO mice showed a significant increase in digging behavior (Fig. 3*J*; Extended Data; Mann–Whitney test; DoF(21); MWU = 16.5; *p* = 0.0013), which can be interpreted as repetitive behavior. We also assessed obsessive–compulsive behaviors in P45–P50 mice using the marble burying test and found that ephrin-B1 KO mice buried significantly more marbles than their controls (Fig. 3*L*; Extended Data; *t* test; *t*_(26)_ = 4.491; *p* < 0.0001). Social behaviors were assessed using three-chamber test and showed reduced sociability and no changes in SNP in ephrin-B1 KO mice compared with controls (Fig. 3*M*,*N*; Extended Data; sociability, *t* test; *t*_(24)_ = 2.758; *p* = 0.0110; SNP, *t* test; *t*_(25)_ = 0.8935; *p* = 0.3801). Anxiety-like behaviors were assessed using the OF test. While control mice increased their exploratory behaviors over time and spent more time in the OF during the second 5 min of the test (Fig. 3*O*; Extended Data; two-way ANOVA; Fisher's exact LSD; genotype, *F*_(1,50)_ = 14.4; *p* = 0.0004; time, *F*_(1,50)_ = 8.338; *p* = 0.0057; interaction, *F*_(1,50)_ = 4.279; *p* = 0.0438; CON first 5 min vs CON second 5 min, *p* = 0.0004), ephrin-B1 KO mice were more anxious and spent less time in the OF during the second 5 min compared with controls (Fig. 3*O*; Extended Data; two-way ANOVA; ephrin-B1 vs CON second 5 min, *p* = 0.0010). There were no significant differences observed in overall distance traveled between the groups (Fig. 5*P*; Extended Data; *t* test; *t*_(25)_ = 0.1259; *p* = 0.9008). Together, the data suggest that impaired inhibition following the deletion of astrocytic ephrin-B1 is most likely responsible for increased susceptibility, duration, and severity of PTZ-induced seizures, as well as exacerbated repetitive and anxiety-like behaviors.

10.1523/JNEUROSCI.0154-24.2024.t3-1 Table 3-1 Statistical analysis for figure 3. Download Table 3-1, DOCX file.

### Astrocytic ephrin-B1 controls localization of EphB in PV boutons but not PV soma

**Figure 4.**
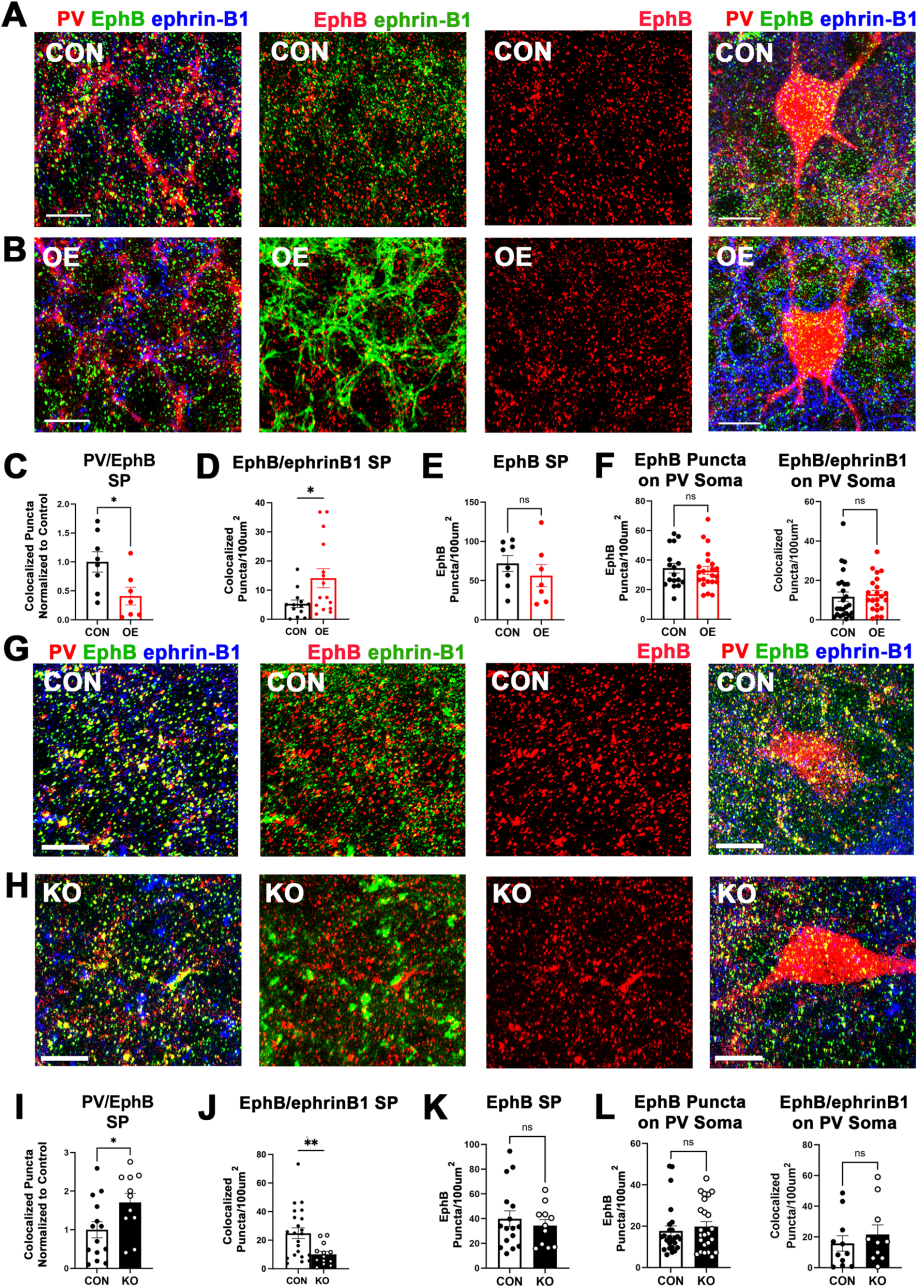
Astrocytic ephrin-B1 levels control localization of EphB in PV boutons but not in the soma of PV interneurons. ***A***, ***B***, ***G***, ***H***, Confocal images from astrocytic ephrin-B1 OE (***B***) and ephrin-B1 KO **(*H*)** and their corresponding controls (CON; ***A***, ***G***) showing PV, pan-EphB, and ephrin-B1 immunofluorescence labeling in the SP layer of CA1 hippocampus and on PV soma. Scale bar, 10 μm. ***C–F***, ***I–L***, Graphs represent the number of colocalized PV/EphB puncta in the SP (***C***, ***I*)**, the number of colocalized EphB/Ephrin-B1 puncta in the SP (***D***, ***J*)**, the total number of EphB puncta in the SP (***E***, ***K***), or the number of EphB puncta and EphB/ephrin-B1 colocalization on PV soma (***F***, ***L*)** in OE mice normalized to the contralateral side of the same brain slice (CON; ***C–F*)** and KO mice normalized to tamoxifen-injected controls lacking ERT2-cre (CON; ***I–L*)**. Although OE (***E***) or deletion (***K***) of astrocytic ephrin-B1 did not affect the total number of EphB puncta in the SP [7–8 images (OE), 11–16 images (KO)/3-4 mice per group; *t* test; *p* > 0.05; Extended Data Table 4-1], the colocalization of EphB with ephrin-B1 and PV boutons was differentially regulated by OE and KO. OE significantly increased EphB/Ephrin-B1 colocalization in the SP (***D***), while KO reduced EphB/Ephrin-B1 colocalization in the SP [***J***; 13–15 images (OE), 13–22 images (KO)/3–4 mice per group; *t* test; *p < 0.05; **p < 0.01; Extended Data Table 4-1]. In contrast, the number of PV-EphB2 colocalized puncta in the SP was significantly reduced in the OE group (***C***) but increased in KO group (***I***) compared with their respective controls [7–8 images (OE); 11–14 images (KO)/3–4 mice per group; *t* test; *p < 0.05; **p < 0.01; Extended Data Table 4-1]. The number of EphB puncta or EphB/ephrin-B1 colocalization on the soma of PV interneurons was unchanged in both OE mice (***F***) and KO mice (***L***; 10-25 PV cells/3–4 mice per group, *t* test, Extended Data Table 4-1). All data are represented as mean ± SEM. Graphics created with Biorender.com.

To test whether astrocytic ephrin-B1 controls PV→PC connectivity through regulating EphB signaling in PV interneurons, we tested if the changes in astrocytic ephrin-B1 levels would affect EphB receptor localization using immunofluorescence staining against PV, pan-EphB, and ephrin-B1 (Fig. 4*A*–*L*). Although neither OE (Fig. 4*E*; Extended Data; *t* test; *t*_(13)_ = 0.9007; *p* = 0.3841) nor deletion (Fig. 4*K*; Extended Data; *t* test; *t*_(25)_ = 0.6443; *p* = 0.5252) of astrocytic ephrin-B1 affected the total number of EphB puncta in the SP layer of the CA1 hippocampus, levels of astrocytic ephrin-B1 influenced the association of the EphB immunoreactive puncta with PV boutons (Fig. 4*C*,*I*) and ephrin-B1 (Fig. 4*D*,*J*). OE of astrocytic ephrin-B1 reduced PV/EphB colocalization in the SP layer compared with the contralateral side of the same brain slice (Fig. 4*C*; Extended Data; *t* test; *t*_(13)_ = 2.503; *p* = 0.0265), showing a significant reduction in the percentage of EphB puncta associated with PV puncta (8.22 ± 1.56 in CON and 5.04 ± 1.74 in OE; *t* test; *p* = 0.046), while deletion of astrocytic ephrin-B1 increased PV/EphB colocalization compared with controls (Fig. 4*I*; Extended Data; *t* test, *t*_(23)_ = 2.246, *p* = 0.0346). In contrast, OE of astrocytic ephrin-B1 increased EphB/ephrin-B colocalization (Fig. 4*D*; Extended Data; Welch-corrected *t* test; *t*_(18.23)_ = 2.5; *p* = 0.0222), while KO reduced EphB/ephrin-B colocalization (Fig. 4*J*; Extended Data; Welch-corrected *t* test; *t*_(30.73)_ = 3.427; *p* = 0.0018). Analysis of EphB receptor on the soma of PV interneurons did not show any significant changes in OE mice (Fig. 4*F*; Extended Data; *t* test; *t*_(37)_ = 0.3604; *p* = 0.7206) or in KO mice (Fig. 4*L*; Extended Data; *t* test; *t*_(47) _= 0.6153; *p* = 0.5413). EphB/ephrin-B1 colocalization on PV somata was also not significantly different in OE (Fig. 4*F*; Extended Data; *t* test; *t*_(45)_ = 0.4070; *p* = 0.6859) or KO mice (Fig. 4*L*; Extended Data; *t* test; *t*_(47)_ = 1.607; *p* = 0.1148) compared with their controls. Altogether, the results show that levels of astrocytic ephrin-B1 did not significantly alter EphB receptor localization on the somata of PV interneurons but instead specifically affected its localization in PV boutons. Reduced levels of PV/EphB colocalization in presynaptic boutons coincided with increased EphB/ephrin-B1 colocalization and PV→PC connectivity in OE mice, while increased PV/EphB colocalization in presynaptic boutons coincided with reduced EphB/ephrin-B1 colocalization and PV→PC connectivity in KO mice. Therefore, we hypothesize that EphB signaling in PV boutons negatively regulates the establishment of inhibitory PV→PC contacts and astrocytic ephrin-B1 controls PV→PC connectivity by regulating the localization of the EphB receptors in PV boutons but not on PV somata.

10.1523/JNEUROSCI.0154-24.2024.t4-1 Table 4-1 Statistical analysis for figure 4. Download Table 4-1, DOCX file.

### Deletion of EphB2 from PV interneurons enhances PV→PC functional connectivity

**Figure 5.**
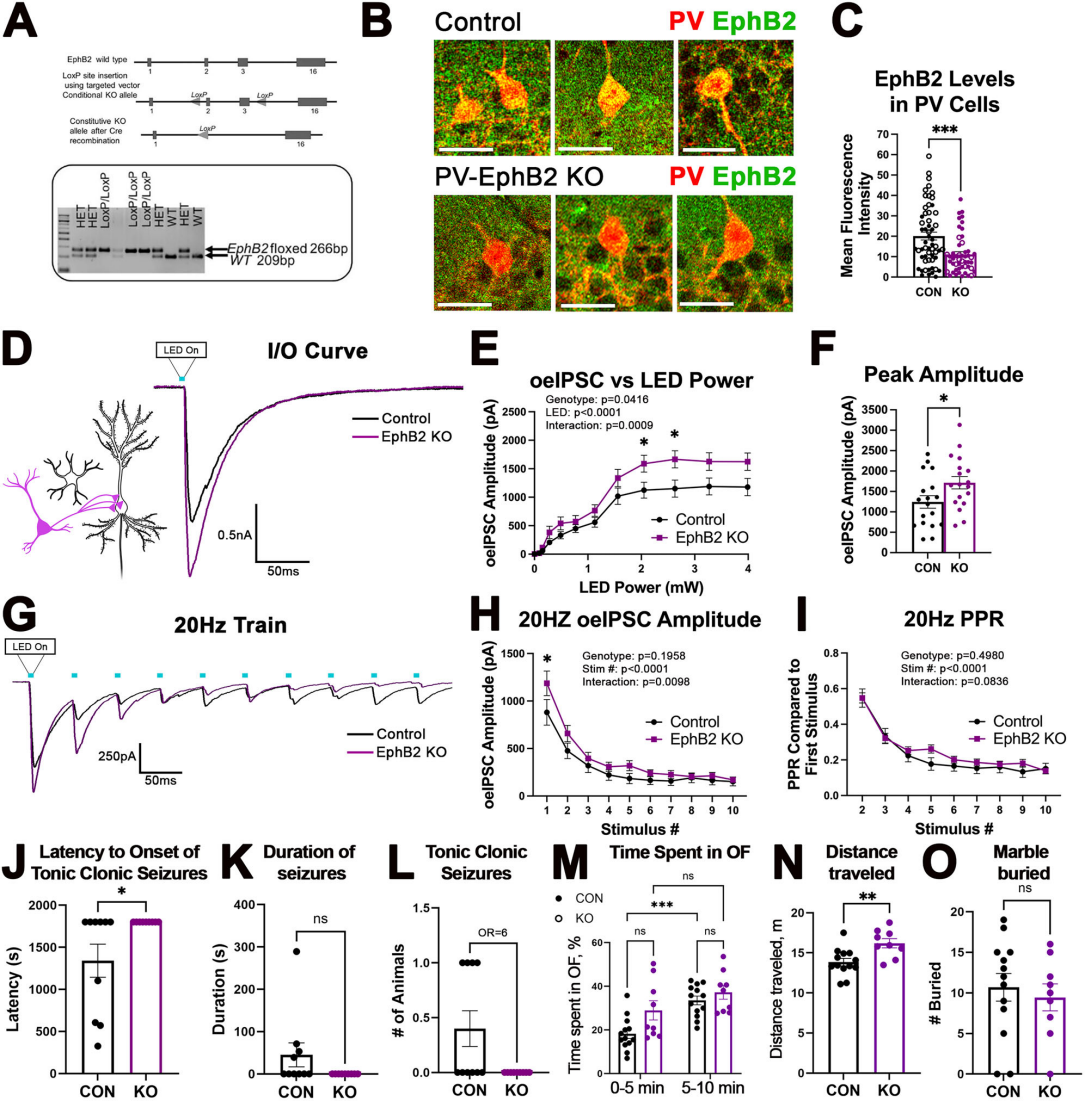
Deletion of EphB2 receptor from PV interneurons enhances PV→PC connectivity and reduces seizure susceptibility. ***A***, Schematics of the generation of EphB2^flox^ mice for conditional deletion of EphB2 using a Cre driver. Exons 2 and 3 were selected as a conditional KO region by inserting the loxP site upstream of Exon 2 and downstream of Exon 3. DNA gel shows the respective genotypes with the WT (209 bp), the heterozygous (HET), and the homozygous EphB2^flox^ (266 bp) bands. ***B***, Confocal images of PV (red) and EphB2 (green) immunolabeling in brain slices from control (top) and PV-EphB2 KO mice (bottom). Scale bar, 25 μm. ***C***, The graph shows quantification of EphB2 immunofluorescence levels in PV interneurons of control and PV-EphB2 KO mice. Deletion of EphB2 from PV interneurons significantly reduced the EphB2 immunofluorescence intensity levels in PV interneurons [*n* = 3–4 mice; 50–57 cells per group; Welch's *t* test; ***p < 0.001; Extended Data Table 5-1). ***D***, Representative current traces of the oeIPSCs recorded from excitatory CA1 PCs of mice lacking EphB2 in PV interneurons (PV-EphB2 KO) and control mice. ***E***, IO curve shows the average oeIPSC amplitudes in PV-EphB2 KO and control mice plotted against LED power. Two-way ANOVA showed a significant effect of genotype, LED power, and interaction between genotype and LED power [18 cells, 7–9 mice per group; two-way ANOVA (sphericity assumed, Sidak's post hoc test); *p < 0.05; Extended Data Table 5-1]. ***F***, The graph shows the average peak oeIPSC amplitude in PV-EphB2 KO and control mice with a significant increase in PV-EphB2 KO group (18 cells; 7–9 mice per group; *t* test; *p < 0.05; Extended Data Table 5-1). ***G***, Representative traces of oeIPSCs from control and PV-EphB2 KO groups during a 20 Hz train of 10 LED pulses. ***H***, The graph shows the average oeIPSC amplitude in control and PV-EphB2 KO cells during each LED pulse within a 20 Hz train. PV-EphB2 KO cells showed a significant increase in the average oeIPSC amplitude during the first stimulus. ***I***, The graph shows the average oeIPSCs normalized to the first stimulus in the 20 Hz train to assess the plasticity with no significant effect of genotype or interaction (18 cells, 7–9 mice per group, two-way ANOVA, sphericity assumed, Sidak's post hoc test, Extended Data Table 5-1). ***J***, The graph shows latency of the onset to tonic–clonic seizure in control and EphB2 KO mice. KO mice showed an increased latency to seizure (9–10 mice per group; Welch-corrected *t* test; *p < 0.05; Extended Data Table 5-1). ***K***, The graph shows the duration of seizure events greater than three, which only occurred in control mice (9–10 mice per group; Welch-corrected *t* test; *p* > 0.05; Extended Data Table 5-1). ***L***, The graph shows the number of animals with tonic–clonic seizures during the test. Four out of ten control mice displayed tonic–clonic seizures, while none of the nine KO mice exhibited tonic–clonic seizures. WT mice were six times more likely to exhibit tonic–clonic seizures than KO mice [odds ratio (OR), 6]. ***M***, ***N***, Graphs show the time spent in OF during the first and second 5 min of the 10 min test and overall distance traveled during the entire 10 min of the test in control and KO mice. ***M***, EphB2 KO mice had more exploratory behaviors with higher overall distance traveled (***N***; 9–13 mice per group; *t* test; **p < 0.01; Extended Data Table 5-1). ***O***, The graph shows marbles buried in the marble burying test, which was not significantly different between control and KO mice. All data are represented as mean ± SEM. Graphics created with Biorender.com.

To test the hypothesis that EphB signaling in PV boutons negatively regulates PV→PC connectivity, we generated a mouse line in which the EphB2 receptor was specifically deleted in PV interneurons. First, EphB2-floxed mice were generated by inserting loxP sites upstream of Exon 2 and downstream of Exon 3 of the *ephB2 gene*. PV-EphB2 KO mice were then generated by crossing PV-Cre and EphB2-floxed mice (Fig. 5*A*). Brain slices from P28 control and PV-EphB2 KO mice were immunolabeled against PV and EphB2 (Fig. 5*B*). We observed a significant reduction in the levels of EphB2 immunoreactivity in PV interneurons (Fig. 5*C*; Extended Data; Welch's *t* test; *t*_(96.88)_ = 3.817; *p* = 0.0002). The presence of residual immunoreactivity may be due to a nonspecific detection of other EphB receptors in PV interneurons. As before, we used whole-cell voltage–clamp electrophysiology in combination with optogenetics to measure PV-specific oeIPSCs in CA1 PCs of control and PV-EphB2 KO mice at P28+/−2D. PV interneurons expressing ChR2-YFP but lacking both copies of EphB2 were optogenetically stimulated using 470 nm LED light, and responses were recorded in CA1 PCs. Deletion of EphB2 from PV interneurons significantly increased the oeIPSC amplitude as a function of increased LED power (Fig. 5*D*,*E*; Extended Data; two-way ANOVA; genotype difference, *F*_(1,34)_ = 4.484; *p* = 0.0416; LED–genotype interaction, *F*_(11,374)_ = 2.946; *p* = 0.0009; Sidak's multiple-comparisons post hoc test; *p < 0.05). The peak oeIPSC amplitude was also significantly increased in PV-EphB2 KO mice compared with controls (Fig. 5*F*; Extended Data; *t* test; *t*_(34)_ = 2.188; *p* = 0.0357). The data indicate that deletion of EphB2 from PV interneurons enhances the strength of PV→PC functional connectivity. The significant enhancement of oeIPSC amplitude was also observed in PV-EphB2 KO mice during the first stimulation in a 20 Hz train (Fig. 5*G*,*H*; Extended Data; two-way ANOVA; genotype difference, *F*_(1,34)_ = 1.741; *p* = 0.195, Stim, *F*_(9,306)_ = 93.98; *p* < 0.0001; interaction, *F*_(9,306)_ = 2.473; *p* = 0.0098; Sidak's multiple-comparison post hoc test; *p < 0.05), but we observed no differences in PPR between the groups (Fig. 5*I*; Extended Data; two-way ANOVA; genotype difference, *F*_(1,34)_ = 0.4691; *p* = 0.4980; Stim, *F*_(8,272)_ = 121.8; *p* < 0.0001; interaction, *F*_(8,272)_ = 1.767; *p* = 0.0836; Sidak's multiple-comparison post hoc test), suggesting that deletion of EphB2 from PV interneurons enhanced functional PV→PC connectivity without changing the presynaptic release probability, most likely due to an increased number or strength of PV→PC synapses.

10.1523/JNEUROSCI.0154-24.2024.t5-1 Table 5-1 Statistical analysis for figure 5. Download Table 5-1, DOCX file.

### Deletion of EphB2 receptors from PV interneurons protects against PTZ-induced seizures but does not alter repetitive or social behaviors

We hypothesized that enhanced PV→PC connectivity should strengthen inhibition in vivo and protect against seizure development. To assess if deletion of EphB2 receptors from PV interneurons influenced seizure susceptibility, we treated EphB2 KO and control mice with PTZ similar to our previous experiments with ephrin-B1 KO mice. However, for this experiment, we utilized a 50 mg/kg dose of PTZ, which was sufficient to induce tonic–clonic seizures in control mice but did not induce tonic–clonic seizures in KO mice (Fig. 5*J–L*). Indeed, KO mice showed a significantly reduced latency to the onset of tonic–clonic seizure (Fig. 5*J*; Extended Data; Welch-corrected *t* test; *t*_(9)_ = 2.335; *p* = 0.0444). While EphB2 KO mice did not experience tonic–clonic seizures, seizure duration was >0 in control mice (Fig. 5*K*; Extended Data; Welch-corrected *t* test; *t*_(8)_ = 1.918; *p* = 0.0914). While 40% of control mice showed tonic–clonic seizures, no events with the score >3 were recorded in KO group, and WT mice were six times more likely to exhibit tonic–clonic seizures than KO mice (Fig. 5*L*; odds ratio, 6). Social behaviors were also assessed in EphB2 KO mice using the three-chamber test which displayed no differences in sociability or social preferences between control and KO mice (data not shown). PV-EphB2 KO mice spent the same amount of time in the OF compared with controls overall, but interestingly PV-EphB2 KO mice showed more exploratory behaviors during the first 5 min (Fig. 5*M*; Extended Data; two-way ANOVA; Tukey's multiple-comparison test; genotype, *F*_(1,40)_ = 6.436; *p* = 0.0152; time, *F*_(1,40)_ = 17.16; *p* = 0.0002; interaction, *F*_(1,40)_ = 1.556; *p* = 0.2195; CON vs KO first 5 min, *p* = 0.0505) and increased distance traveled than controls during all 10 min of the test (Fig. 5*N*; Extended Data; *t* test; *t*_(20)_ = 3.213; *p* = 0.0044). We observed no changes in repetitive behaviors, as KO mice buried the same number of marbles compared with controls (Fig. 5*O*; Extended Data; *t* test; *t*_(20)_ = 0.5060; *p* = 0.6184). The data suggest that deletion of EphB2 from PV interneurons protects against PTZ-induced seizures and enhances exploratory behaviors, but does not alter repetitive or social behaviors.

### Deletion of EphB2 receptors from PV interneurons enhances PV→PC structural connectivity

**Figure 6.**
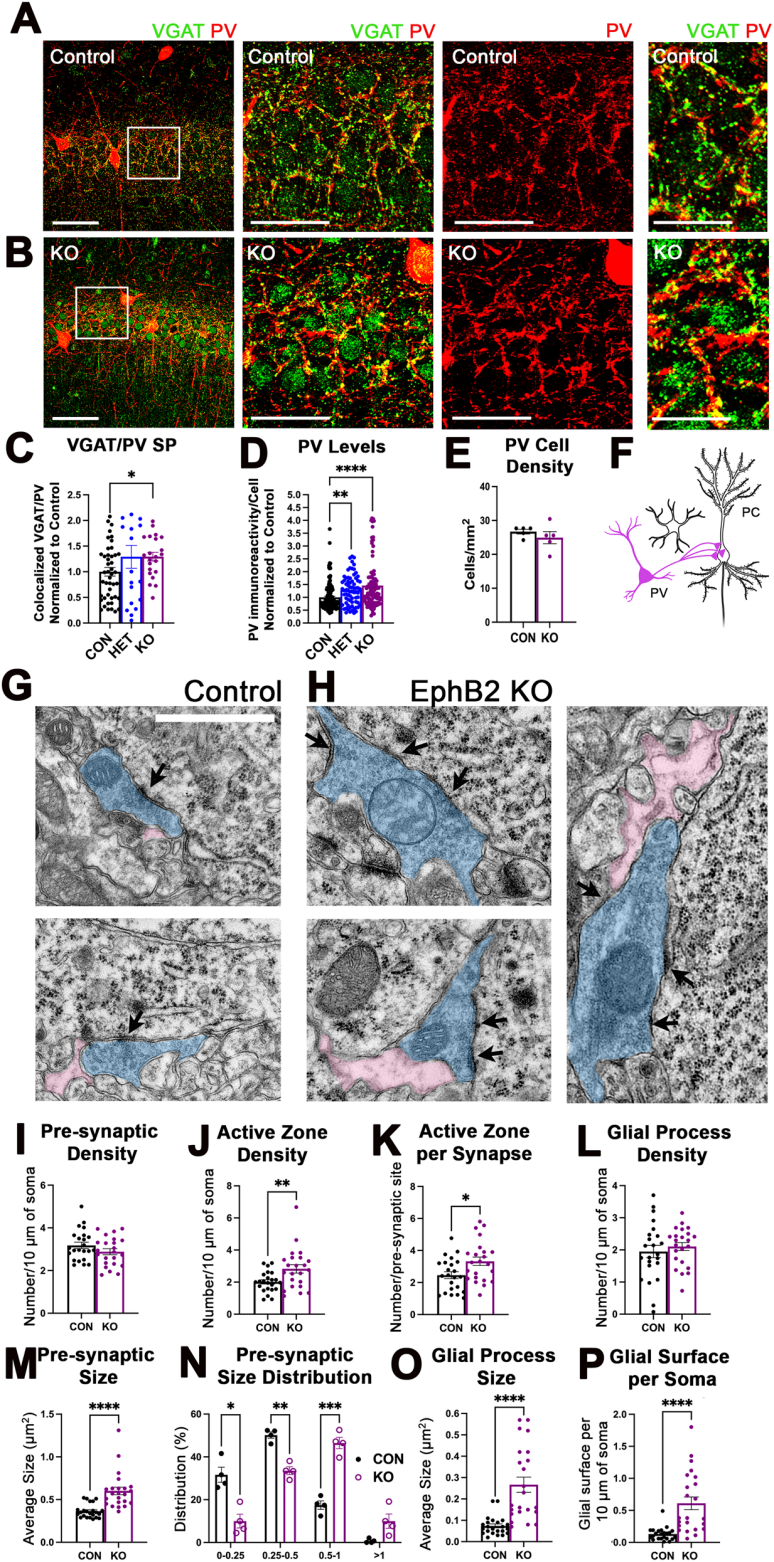
Deletion of EphB2 receptors from PV interneurons enhances PV→PC structural connectivity and perisomatic innervation. ***A***, ***B***, Confocal images of brain slices from (***A***) control and (***B***) PV-EphB2 KO immunolabeled against VGAT (green) and PV (red); scale bar, 50 μm (left panels), 25 μm (middle panels), and 10 μm (right panels). ***C***, The graph shows the average number of colocalized VGAT/PV puncta in the SP layer of the CA1 hippocampus of control, PV-EphB2^+/−^, and PV-EphB2^−/−^ KO mice normalized to control. PV-EphB2^−/−^ KO mice showed a significant increase in the number of VGAT/PV colocalized puncta compared with controls (19–46 images/5–13 mice per group; Brown–Forsythe and Welch ANOVA test; Dunnett's multiple-comparison test; *p < 0.05; Extended Data Table 6-1). ***D***, The graph shows the average PV immunoreactivity in control, PV-EphB2^+/−^, and PV-EphB2^−/−^ KO mice normalized to control. PV immunoreactivity was increased in mice lacking one or both copies of EphB2 (72–165 cells/group; 4–13 mice/group; Brown–Forsythe and Welch ANOVA test; Dunnett's multiple-comparison test; **p < 0.01; ****p < 0.0001; Extended Data Table 6-1). ***E***, The graph shows average PV cell density in control and PV-EphB2^−/−^ KO mice. There were no significant differences in the number of PV cells detectable by immunostaining (15 images/5 mice per group; *t* test; *p* > 0.05; Extended Data Table 6-1). ***F***, The graphic shows the changes in PV→PC connectivity in PV-EphB2 KO mice. ***G***, ***H***, Electron microscope images of the CA1 SP layer of (***G***) control and (***H***) PV-EphB2^−/−^ KO mice taken at 8,000×. Scale bar, 1 μm. ***I–L***, Graphs show the density of perisomatic presynaptic sites (***I***), active zone density (***J***), the number of active zones per presynaptic bouton (***K***), and the density of glial processes (***L***). The density of perisomatic presynaptic sites and glial processes was not changed following EphB2 KO in PV cells (23 images/3 mice per group; *t* test; *p* > 0.05; Extended Data Table 6-1). However, the number of active zones was significantly increased in KO mice (23 images/3 mice per group; *t* test; *p < 0.05; **p < 0.01; Extended Data Table 6-1). ***M–P***, Graphs show the average size of perisomatic presynaptic sites (***M***), presynaptic size distribution (***N***), average size of glial processes (***O***), and glial surface per neuronal soma (***P***) in control and KO mice. EphB2 KO mice showed an increased average size of presynaptic boutons (23 images/3 mice per group; *t* test; ****p < 0.0001; Extended Data Table 6-1) and a higher number of larger presynaptic sites (23 images/3 mice per group; 2-way ANOVA; Sidak multiple-comparison test; *p < 0.05; **p < 0.01; ***p < 0.001; Extended Data Table 6-1). EphB2 KO also showed a significantly larger size of glial processes and the area of glial surface per neuronal soma (23 images/3 mice per group; *t* test; ****p < 0.0001; Extended Data Table 6-1). All data are represented as mean ± SEM. Graphics created with Biorender.com.

To test if levels of EphB2 in PV interneurons affect the number of PV-positive inhibitory synapses during P14–P28 period, we analyzed the density of PV-positive inhibitory presynaptic sites in the pyramidal (SP) layer of the CA1 hippocampus by immunolabeling hippocampal slices against PV and VGAT (Fig. 6*A*,*B*). Mice lacking both copies of EphB2 in PV interneurons showed a significant increase in the number of VGAT/PV presynaptic sites in the SP compared with controls, while deletion of one copy of EphB2 in PV interneurons had no significant effect (Fig. 6*C*; Extended Data; Brown–Forsythe and Welch ANOVA; *F*_(2.0,32.24)_ = 1.837; *p* = 0.1756; Dunnett's multiple-comparison test, CON-HET, *p* = 0.5407; CON-KO, *p* = 0.0486; *p < 0.05). Deletion of one or both copies of EphB2 in PV interneurons was also sufficient to increase PV immunoreactivity in PV interneurons (Fig. 6*D*; Extended Data; Brown–Forsythe and Welch ANOVA; *F*_(2.0,202.7)_ = 13.5; *p* < 0.0001; Dunnett's multiple-comparisons test; CON-HET, *p* = 0.0012; CON-KO, *p* < 0.0001; **p < 0.01; ****p < 0.0001). However, there were no differences in the density of PV interneurons detectable by immunostaining (Fig. 6*E*; Extended Data; *t* test; *t*_(8)_ = 0.9220; *p* = 0.3835). As PV expression is activity dependent and thought to correlate with PV cell activity and maturation (Donato et al., 2013; Page et al., 2019), PV interneuron activity or maturation may be negatively regulated by EphB2 expression in PV interneurons.

10.1523/JNEUROSCI.0154-24.2024.t6-1 Table 6-1 Statistical analysis for figure 6. Download Table 6-1, DOCX file.

In addition, we utilized EM to analyze the number of perisomatic presynaptic sites in the CA1 SP layer of control and PV-EphB2 KO mice, which resembled inhibitory presynaptic sites with large presynaptic vesicles and adjacent symmetric Type 2 axosomatic synapses (Fig. 6*G*,*H*). Although we did not detect any significant differences in the density of presynaptic sites on neuronal somata (Fig. 6*I*; Extended Data; *t* test; *t*_(44)_ = 1.447; *p* = 0.1551), both the density of active zones (Fig. 6*J*; Extended Data; Welch-corrected *t* test; *t*_(32.55)_ = 2.767; *p* = 0.0092) and the number of active zones per presynaptic site were increased in KO mice (Fig. 6*K*; Extended Data; *t* test; *t*_(44)_ = 2.539; *p* = 0.0147). The average size of the presynaptic site was also significantly higher in KO mice (Fig. 6*M*; Extended Data; *t* test; *t*_(44)_ = 5.012; *p* < 0.0001). KO mice showed a higher proportion of presynaptic sites of a larger size (0.5–1 μm) and a lower proportion of smaller presynaptic sites (0–0.5 μm; Fig. 6*M*; Extended Data; ANOVA with Tukey's multiple-comparison test; *F*_(7,24)_ = 52; for 0–0.25 *p* < 0.0001; for 0.25–0.5 *p* = 0.002; for 0.5–1 *p* < 0.0001; for >1 *p* = 0.2010). Interestingly, the size of glial processes (Fig. 6*O*; Extended Data; Welch-corrected *t* test; *t*_(24.91)_ = 5.226; *p* < 0.0001) as well as coverage of neuronal somata was also enhanced in KO mice (Fig. 6*P*; Extended Data; Welch-corrected *t* test; *t*_(23.97)_ = 4.721; *p* < 0.0001). However, the total number of glial processes was unchanged (Fig. 6*L*; Extended Data; *t* test; *t*_(44)_ = 0.6854; *p* = 0.4967). The increased size of inhibitory presynaptic sites may explain our ability to detect an increased number of VGAT/PV presynaptic sites in KO mice and the increase in the number of active zones within inhibitory–presynaptic sites may explain why we detect enhanced strength of PV–PC functional connections in KO mice. Altogether the results suggest that EphB2 signaling in PV interneurons may negatively regulate PV–PC connectivity through controlling the size of inhibitory presynaptic sites and number of active zones within them. This is potentially achieved by regulating the recruitment of glial processes.

### Astrocytic ephrin-B1 OE does not further enhance PV→PC connectivity in mice lacking EphB2 in PV interneurons

**Figure 7.**
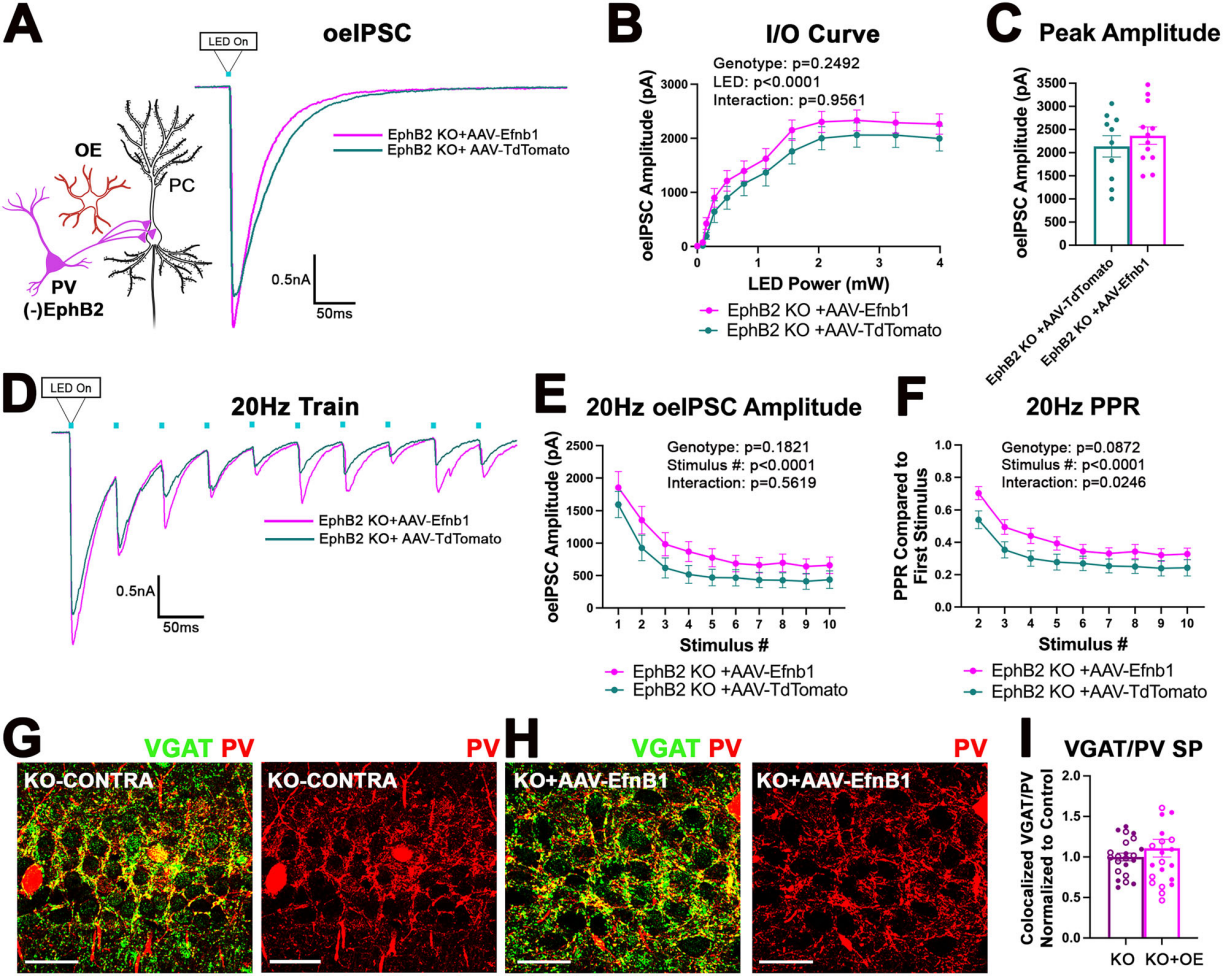
Astrocytic ephrin-B1 OE does not further enhance PV→PC connectivity in mice lacking EphB2 in PV interneurons. ***A***, Representative current traces of oeIPSCs recorded from excitatory CA1 PCs of mice overexpressing astrocytic ephrin-B1 but lacking EphB2 in PV interneurons (PV-EphB2 KO+AAV-EfnB1) and vehicle-injected mice lacking EphB2 in PV interneurons (PV-EphB2 KO+AAV-TdT). ***B***, The IO curve shows the average oeIPSC amplitude in PV-EphB2 KO+AAV-EfnB1 and in PV-EphB2 KO+AAV-TdT mice plotted against LED power. OE of astrocytic ephrin-B1 in mice lacking EphB2 in PV interneurons did not significantly change the oeIPSC amplitude compared with control mice [*n* = 10–12 cells, 5–6 mice per group, 2-way ANOVA (sphericity not assumed, Sidak post hoc test), Extended Data Table 7-1]. ***C***, The graph shows the average peak amplitude achieved during generation of the IO curve. oeIPC amplitudes recorded in CA1 PCs of PV-EphB2 KO mice overexpressing astrocytic ephrin-B1 were not significantly different from those recorded in PCs of PV-EphB2 KO mice injected with control AAV-TdT (*n* = 11–12 cells, 5–6 mice per group, *t* test, Extended Data Table 7-1). ***D***, Representative traces of the oeIPSCs from PV-EphB2 KO+AAV-EfnB1 and PV-EphB2 KO + AAV-TdT cells, generated during stimulation with a 20 Hz train of 10 LED pulses. ***E***, The graph shows the average oeIPSC amplitude in PV-EphB2 KO+AAV-EfnB1 and PV-EphB2 KO+AAV-TdT cells during each LED pulse within a 20 Hz train. There was no significant genotype or interaction effect. ***F***, The graph shows the average oeIPSCs normalized to the first stimulus in the 20 Hz train to assess the plasticity with no significant effect of genotype; however, there was a significant interaction effect (***E***, ***F***, 9–10 cells, 5–6 mice per group, 2-way ANOVA, Extended Data Table 7-1). ***G***, ***H***, Confocal images of brain slices from (***H***) EphB2 KO+AAV-EfnB1 mice (KO+OE) and (***G***) the contralateral noninjected side (KO) of the same brain slice immunolabeled against VGAT (green) and PV (red); scale bar, 25 μm. (***I***) The graph shows the number of VGAT/PV colocalized puncta in the SP normalized to the contralateral side of the same brain slice. OE of astrocytic ephrin-B1 in mice lacking EphB2 in PV interneurons did not affect the number of VGAT/PV presynaptic sites (22 images/6 mice per group, *t* test, Extended Data Table 7-1). All data are represented as mean ± SEM. Graphics created with Biorender.com.

If astrocytic ephrin-B1 regulates PV→PC connectivity through controlling EphB2 signaling in PV boutons, we expect that OE of astrocytic ephrin-B1 in mice lacking EphB2 in PV interneurons should not further enhance PV→PC connectivity. To test this hypothesis, we performed whole-cell voltage–clamp electrophysiology recordings to measure PV-specific oeIPSCs in CA1 PCs of PV-EphB2 KO mice injected with AAV-EfnB1 to overexpress ephrin-B1 in astrocytes or AAV-TdTomato control vector. PV interneurons were optogenetically stimulated, and responses were recorded in CA1 PCs. IO curves were generated as described previously and maximum responses were used to measure peak oeIPSCs (Fig. 7*A*–*C*). We observed a significant effect of LED power but no effects of genotype or interaction between genotype and LED power (Fig. 7*B*; Extended Data; two-way ANOVA; genotype, *F*_(1,20)_ = 1.409; *p* = 0.2492; LED power, *F*_(2.460,49.19)_ = 104.1; *p* < 0.0001; interaction, *F*_(11,220)_ = 0.3973; *p* = 0.9561; Sidak's multiple-comparison post hoc test). There was also no significant difference in the peak oeIPSC amplitude between the groups (Fig. 7*C*; Extended Data; *t* test; *t*_(20)_ = 0.7851; *p* = 0.4461). The data suggest that astrocytic ephrin-B1 OE did not further enhance the strength of PV→PC functional connections in ephrin-B1–injected PV-EphB2 KO compared with control-injected PV-EphB2 KO group. OE of astrocytic ephrin-B1 in mice lacking EphB2 in PV interneurons also did not affect oeIPSC amplitude over a 20 Hz train (Fig. 7*E*; Extended Data; two-way ANOVA; genotype, *F*_(1,17)_ = 1.936; *p* = 0.1821; Stim, *F*_(9,153)_ = 96.12; *p* < 0.0001; interaction, *F*_(9,153)_ = 0.8606; *p* = 0.5619; Sidak's multiple-comparison post hoc test). Interestingly, there was a trend toward increased PPR and a significant interaction between stimulus number and genotype (Fig. 7*F*; Extended Data; two-way ANOVA; genotype, *F*_(1,17)_ = 3.294; *p* = 0.0872; Stim, *F*_(2.958,50.28)_ = 102.3; *p* < 0.0001; interaction, *F*_(8,136)_ = 2.293; *p* = 0.0246; Sidak's multiple-comparison post hoc test), suggesting that the two groups may exhibit differences in short-term synaptic dynamics. Analysis of structural VGAT/PV presynaptic sites by immunolabeling also did not reveal any significant difference between ephrin-B1 overexpressing PV-EphB2 KO and the contralateral control side of the same brain slice (Fig. 7*I*; Extended Data; paired *t* test; *t*_(21)_ = 0.9448; *p* = 0.3555). These data support the hypothesis that astrocytic ephrin-B1 controls PV→PC connectivity by negatively regulating EphB receptor signaling in PV boutons, allowing for more inhibitory connections to form between PV and PC cells.

10.1523/JNEUROSCI.0154-24.2024.t7-1 Table 7-1 Statistical analysis for figure 7. Download Table 7-1, DOCX file.

**Figure 8.**
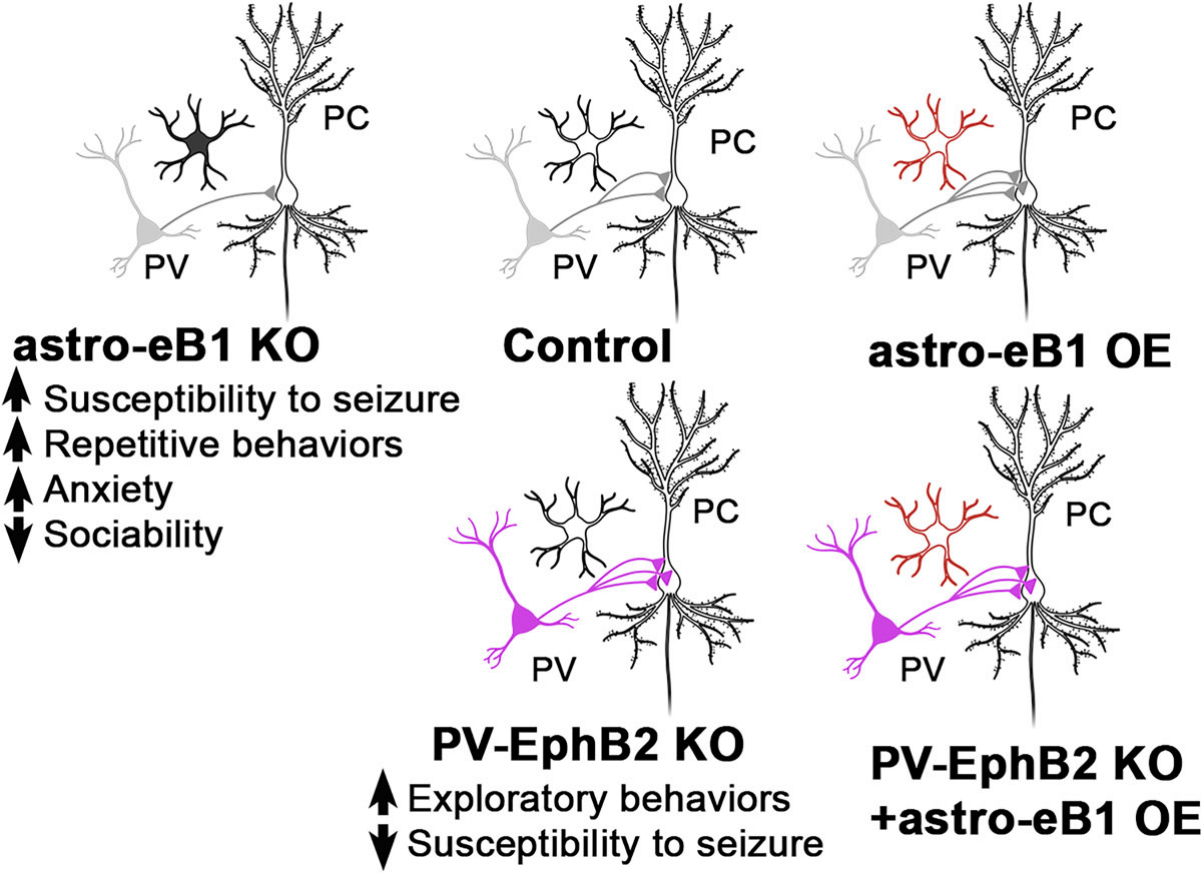
Astrocytes control PV→PC connectivity and inhibition in vivo through regulating EphB2 signaling in PV boutons: deletion of astrocytic ephrin-B1 impaired PV→PC structural connectivity and impaired inhibition in vivo, leading to increased seizure susceptibility, deficits in sociability, anxiety-like behaviors, and repetitive behaviors. Conversely, OE of astrocytic ephrin-B1 increased PV→PC structural and functional connectivity. Deletion of EphB2 receptor in PV cells also increased PV→PC structural and functional connectivity, resulting in reduced seizure susceptibility and increased exploratory behaviors, indicative of reduced anxiety. Deletion of astrocytic ephrin-B1 in mice lacking EphB2 in PV cells did not lead to further enhancement of PV→PC structural or functional connectivity, suggesting that astrocytic ephrin-B1 controls PV→PC connectivity through a mechanism involving EphB2 signaling in PV cells.

## Discussion

Here we report three major findings: (1) genetic deletion of EphB2 receptor from PV cells during early postnatal hippocampal development enhances PV cell structural and functional connectivity; (2) OE of EphB2 receptor ligand, ephrin-B1, in astrocytes affects EphB2 localization in PV-positive inhibitory perisomatic boutons and promotes PV cell activity; and (3) the effects of astrocytic ephrin-B1 on PV cell structural and functional connectivity are mediated through EphB2 receptor signaling in PV cells (Fig. 8). More specifically, we show that the strength of PV→PC connectivity was affected by the levels of ephrin-B1 in astrocytes. OE of ephrin-B1 in astrocytes led to a significant increase in the amplitude of PV-specific oeIPSCs recorded in PCs with no significant difference in the PPR. The enhancement of oeIPSC amplitude following OE of astrocytic ephrin-B1 can be explained by an increased number of synapses or increased GABA receptor number or function. Although activation of the EphB receptor with its ligand ephrin-B was reported to regulate AMPAR and NMDAR clustering and function, its effects on GABA receptors have not been observed (Dalva et al., 2000; Takasu et al., 2002; Kayser et al., 2006). In addition, the analysis of mRNA levels of multiple GABA receptors showed no changes in their levels in ephrin-B1 KO mice (data not shown). Our findings suggest that astrocytic ephrin-B1 negatively regulates EphB receptor signaling at the presynaptic site of inhibitory synapses, as we observe an increased number of VGAT/PV presynaptic sites following astrocytic ephrin-B1 OE as well as PV-specific deletion of EphB2. In contrast, the reduction in VGAT/PV presynaptic sites in ephrin-B1 KO coincided with a decrease in VGAT/gephyrin inhibitory synapses on PC somata in the CA1 hippocampus, suggesting that the observed changes in PV-positive presynaptic sites are most likely responsible for reduced inhibition. The observations that both strength and number of PV→PC synapses are regulated by the levels of ephrin-B1 in astrocytes and EphB2 in PV cells provide further evidence that the development of PV-positive inhibitory synapses is tightly regulated by astrocytes through EphB receptor signaling in PV cells.

*E*/*I* imbalance is hypothesized to drive pathological phenotypes exhibited in NDDs (Rubenstein and Merzenich, 2003). More specifically, impaired inhibition is thought to underlie the development of hyperactive neuronal networks in NDDs such as ASD and epilepsy (Sohal and Rubenstein, 2019; Tang et al., 2021). Dysfunctions of PV interneurons, including reduced density of PV interneurons, reduced PV expression, impaired PNN formation, and reduced activity of PV interneurons have been observed in individuals with ASD, suggesting that PV interneurons may critically regulate *E*/*I* balance and that they may play an important role in the pathogenesis of NDDs (Lawrence et al., 2010; Hashemi et al., 2016; Contractor et al., 2021). Our data show that deletion of astrocytic ephrin-B1 increases susceptibility to PTZ-induced seizures, but deletion of EphB2 in PV interneurons reduces seizure susceptibility, which provide further evidence of the involvement of ephrin-B/EphB signaling in regulating inhibition in vivo. We speculate that the reduction in PV→PC connectivity most likely contributes to this impaired inhibition. However we previously reported that the deletion of astrocytic ephrin-B1 increased the number of excitatory synapses in the CA1 hippocampus, which can also contribute to the enhanced seizure susceptibility in ephrin-B1 KO mice (Nguyen et al., 2020). We also found that mice lacking ephrin-B1 in astrocytes exhibit repetitive digging behavior and increased marble burying compared with controls. Astrocyte-specific deletion of ephrin-B1 also enhances anxiety-like behaviors in the OF test, and juvenile P45–P50 ephrin-B1 KO mice also exhibit impaired sociability in the three-chamber test similar to P28 mice as previously reported (Nguyen et al., 2020). In contrast, we find that the deletion of EphB2 in PV interneurons had no effect on sociability (results not shown) or marble burying but did enhance exploratory behaviors in the OF test, which implicates that the mice were less anxious. Impaired social behaviors and restrictive, repetitive behaviors are currently two of the major diagnostic criteria for ASD (Association, 2013). Additionally, ASD individuals present with epilepsy at disproportionate rates compared with the normal population (Spence and Schneider, 2009; Tuchman and Cuccaro, 2011). Importantly, de novo mutations in the gene encoding EphB2 receptors have been reported to be risk factors for the development of ASD, and EphB2 is identified as a strong candidate with Score 2 in SFARI database (Kong et al., 2012; Sanders et al., 2012; Abrahams et al., 2013; Yang et al., 2013). Therefore, we believe the results of our study illustrate a novel mechanism responsible for proper inhibitory circuit development, which when impaired can result in ASD phenotypes.

We found that levels of astrocytic ephrin-B1 influence the localization of EphB receptor clusters in PV boutons but not on the somata of PV interneurons. It is intriguing that the levels of astrocytic ephrin-B1 influence the localization of EphB receptors in a compartment-specific manner. The compartment-specific nature of this regulation is a rather significant finding, as presynaptic versus postsynaptic EphB signaling may regulate diverse and/or opposing functions in neuronal development and synaptic maintenance. For example, postsynaptic EphB receptors play important roles in recruiting and stabilizing AMPA/NMDARs in excitatory postsynaptic membranes in dendrites and also regulate dendritic filopodial motility, spine morphogenesis, and dendritic spine maturation, thereby coordinating excitatory synaptic development (Dalva et al., 2000; Ethell et al., 2001; Takasu et al., 2002; Kayser et al., 2006; Sloniowski and Ethell, 2012). If EphB2 is also involved in regulating excitatory input onto PV cells, then we would expect deletion of EphB2 from PV cells to impair excitatory synapse development onto PV cells instead of enhancing it. However, we observe the opposite effect as EphB2 deletion potentiates PV activity and PV→PC connectivity. As we saw the most robust effects of astrocytic ephrin-B1 on the localization of the EphB receptor in PV boutons, we believe our work suggests that astrocytic ephrin-B1 regulates PV→PC connectivity by limiting the repulsive trans-synaptic interactions between EphB expressing PV boutons and ephrin-B expressing soma or axon initial segment (AIS) of PCs. Additionally, since astrocytic ephrin-B1 negatively regulated excitatory synapse development (Nguyen et al., 2020), we would expect ephrin-B1 KO in astrocytes to increase clustering of NMDAR and AMPAR in excitatory PC→PV synapses, enhancing excitatory drive onto PV interneurons and increasing inhibition. However, we see a decrease in inhibition of PCs following ephrin-B1 KO in astrocytes, supporting our hypothesis that EphB expression in PV boutons but not somata of PV interneurons regulates their synaptic connectivity.

Here we propose a new mechanism of astrocyte-mediated regulation of inhibitory synapse assembly involving EphB receptor signaling in PV-positive presynaptic boutons. Our observations that a reduction in EphB receptor association with PV boutons following the OE of astrocytic ephrin-B1 or genetic deletion of EphB2 from PV interneurons both enhances the assembly of PV presynaptic sites are consistent with the adverse role of EphB receptor in inhibitory synapse development. To examine the role of EphB signaling in PV interneurons, we used PV-cre promoter to drive cre-lox–mediated deletion of floxed EphB2 receptors. As ephrin-B/EphB signaling has also been implicated in the migration of Dlx1/2 expressing inhibitory interneurons during embryonic development (Talebian et al., 2017), it is important to note that PV expression begins ∼P10–12 and gradually increases until about the third postnatal week (Seto-Ohshima et al., 1990; de Lecea et al., 1995; Yang et al., 2013), timing EphB2 deletion during the P14–P21 period, well after PV interneuron migration to the hippocampus occurs (Tricoire et al., 2011; Pelkey et al., 2017). If EphB2 plays a role in promoting excitatory innervation of PV interneurons, we would expect reduced activity of PV interneurons following its deletion. However, similar to overexpressing astrocytic ephrin-B1, we found that deletion of EphB2 from PV boutons increases PV-specific oeIPSCs recorded in PCs. Our findings suggest that EphB2 negatively regulates PV→PC contact formation and most likely acts as a repulsive cue for inhibitory synapse development. Our hypothesis is supported by the fact that disrupting trans-synaptic ephrin-B/EphB2 interactions by removing EphB2 is sufficient to trigger an increase in PV→PC connectivity. This is also in line with other published work showing that ectopic expression of EphB4 in PV interneurons reduced perisomatic innervation by PV interneurons in the visual cortex (Baohan et al., 2016). Interestingly, this group found that under normal conditions, Pten suppresses EphB4 expression but that loss of a single copy of Pten increased EphB4 expression and impaired PV→PC connection probability, without affecting PC→PV connectivity. This is consistent with our finding that EphB receptor levels are regulated in PV boutons and are important for PV→PC connectivity but may be less important for PC→PV connectivity (Baohan et al., 2016). We hypothesized that astrocytic ephrin-B1 regulated PV→PC connectivity by impairing repulsive trans-synaptic ephrin-B/EphB signaling. Consistent with our hypothesis, we found that OE of ephrin-B1 in astrocytes did not further enhance PV→PC connectivity in mice lacking EphB2 in PV interneurons. Interestingly, it has been shown that EphB2 signaling kinetics dictate whether or not a stable contact will form so that large amounts of EphB2 signaling very quickly will mediate repulsion; however, slower, more gradual EphB2 signaling facilitates adhesion (Mao et al., 2018). It is possible that removal of one EphB receptor or interruption of some but not all trans-synaptic ephrin-B/EphB signaling by astrocytes works through a mechanism that simply slows down the EphB signaling kinetics within the PV bouton, encouraging contact formation. Astrocytes could regulate EphB2 signaling in PV boutons in a number of ways. For example, it has been shown that astrocytes are able to transendocytose neuronal EphB2 in a manner dependent on astrocytic ephrin-B1 (Lauterbach and Klein, 2006). Therefore, astrocytes may be able to transendocytose and remove neuronal EphB2 in vivo, limiting EphB2 signaling in PV boutons and enhancing PV→PC connectivity. Additionally, ephrin/EphB2 binding can induce EphB receptor cleavage through matrix metalloproteinases (MMP) to terminate EphB signaling (Lin et al., 2008). Astrocytic ephrin-B1 could neutralize EphB signaling in PV boutons through MMP-mediated receptor cleavage. Future studies should investigate how astrocytes are regulating neuronal EphB signaling in PV interneurons.

In summary, we show that astrocytes utilize ephrin-B1 to regulate PV→PC connectivity through interfering with repulsive EphB signaling in PV interneurons. Our study implicates EphB2 signaling as an important developmental cue for inhibitory synapse development between PV interneurons and PCs. Disruption of this regulatory mechanism leads to impaired inhibition in vivo as evidenced by increased seizure susceptibility and the presence of repetitive behaviors. As PV interneuron dysfunction is implicated in the pathogenesis of ASD (Rubenstein and Merzenich, 2003; Ali Rodriguez et al., 2018; Sohal and Rubenstein, 2019; Contractor et al., 2021) and EphB2 has been reported to be a strong risk factor for the development of ASD (Sanders et al., 2012), EphB signaling in PV interneurons can also serve as an important and powerful therapeutic target to correct impaired inhibition in NDDs. Future studies can address whether aberrant EphB2 signaling in PV interneurons underlies the development of pathological ASD phenotypes in NDDs.
