# Supplementary material for: EphB2 Signaling Is Implicated in Astrocyte-Mediated Parvalbumin Inhibitory Synapse Development
Source: J Neurosci. 2024 Sep 26;44(45):e0154242024. doi: 10.1523/JNEUROSCI.0154-24.2024 (PMC11551896; doi:10.1523/JNEUROSCI.0154-24.2024)
Supplement: Table 1-1 — Statistical analysis for figure 1. Download Table 1-1, DOCX file. [file jneuro-44-e0154242024-s001.docx]

Extended Data Fig. 1D

|  | Mean | SEM | N |
| --- | --- | --- | --- |
| Control | 1.000 | 0.0875 | 12 |
| KO | 0.3923 | 0.1143 | 8 |
| Statistics | t=4.280, df=18, p=0.0005 |  |  |

Extended Data Fig. 1E

SP

|  | Mean | SEM | N |
| --- | --- | --- | --- |
| CON | 6.754 | 0.3573 | 61 |
| KO | 6.371 | 0.2874 | 62 |
| Statistics | t=0.8369, df=121, p=0.4043 |  |  |

All Layers

|  | Mean | SEM | N |
| --- | --- | --- | --- |
| CON | 16.33 | 0.7617 | 61 |
| KO | 15.56 | 0.6519 | 62 |
| Statistics | t=0.7623, df=121, p=0.4474 |  |  |

Extended Data Fig. 1G

|  | Mean | SEM | N |
| --- | --- | --- | --- |
| CON | 0.9750 | 0.0372 | 11 |
| OE (AAV-EfnB1) | 1.828 | 0.2444 | 11 |
| Statistics | t=3.629, df=10, p=0.0046 |  |  |

Extended Data Fig. 1H

SP

|  | Mean | SEM | N |
| --- | --- | --- | --- |
| CON | 0.1572 | 0.0556 | 14 |
| OE | 0.3487 | 0.1202 | 15 |
| Statistics | t=1.412, df=27, p=0.1695 |  |  |

SO

|  | Mean | SEM | N |
| --- | --- | --- | --- |
| CON | 0.1666 | 0.03779 | 14 |
| OE | 0.1877 | 0.02825 | 15 |
| Statistics | t=0.4519, df=27, p=0.6549 |  |  |

Extended Data Fig. 1K

|  | **CON (TdTomato-AAV)** | | | **OE (AAV-EfnB1)** | | |
| --- | --- | --- | --- | --- | --- | --- |
|  | **Mean** | **SEM** | **N** | **Mean** | **SEM** | **N** |
| Row 1 | 13.97836364 | 8.851988356 | 11 | 9.99972225 | 9.99972 | 12 |
| Row 2 | 71.66554545 | 25.16898757 | 11 | 156.204917 | 68.9252 | 12 |
| Row 3 | 364.3725 | 107.090452 | 11 | 410.612292 | 122.296 | 12 |
| Row 4 | 682.3684091 | 132.5836899 | 11 | 701.769583 | 148.773 | 12 |
| Row 5 | 922.7524243 | 132.7234792 | 11 | 1044.56444 | 173.727 | 12 |
| Row 6 | 1098.870909 | 141.0408263 | 11 | 1281.60208 | 189.901 | 12 |
| Row 7 | 1249.734848 | 147.4226701 | 11 | 1602.83236 | 141.93 | 12 |
| Row 8 | 1586.656515 | 155.8329254 | 11 | 2082.98611 | 124.406 | 12 |
| Row 9 | 1664.102727 | 163.3410148 | 11 | 2117.76111 | 127.46 | 12 |
| Row 10 | 1677.693333 | 160.3151056 | 11 | 2095.69861 | 120.498 | 12 |
| Row 11 | 1674.138788 | 158.1509816 | 11 | 2115.5875 | 140.67 | 12 |
| Row 12 | 1596.158182 | 136.2065692 | 11 | 2085.50972 | 147.579 | 12 |

| **ANOVA table** | **SS** | **DF** | **MS** | **F (DFn, DFd)** | **P value** | **% of total variation** |
| --- | --- | --- | --- | --- | --- | --- |
| LED power x genotype | 2534443 | 11 | 230404 | F (11, 231) = 2.684 | P=0.0029 | 1.302 |
| LED power | 134239662 | 11 | 1.2E+07 | F (11, 231) = 142.2 | P<0.0001 | 68.95 |
| genotype | 4603906 | 1 | 4603906 | F (1, 21) = 3.049 | P=0.0954 | 2.365 |
| cell | 31714493 | 21 | 1510214 | F (21, 231) = 17.59 | P<0.0001 | 16.29 |

| **Šídák's multiple comparisons test** | **Predicted (LS) mean diff.** | **95.00% CI of**  **diff.** | **Adjusted P Value** |
| --- | --- | --- | --- |
| CON - OE |  |  |  |
| Row 1 | 3.979 | -540.5 to 548.5 | >0.9999 |
| Row 2 | -84.54 | -629.0 to 459.9 | >0.9999 |
| Row 3 | -46.24 | -590.7 to 498.2 | >0.9999 |
| Row 4 | -19.4 | -563.9 to 525.1 | >0.9999 |
| Row 5 | -121.8 | -666.3 to 422.7 | 0.9998 |
| Row 6 | -182.7 | -727.2 to 361.7 | 0.9924 |
| Row 7 | -353.1 | -897.6 to 191.4 | 0.5396 |
| Row 8 | -496.3 | -1041 to 48.14 | 0.1038 |
| Row 9 | -453.7 | -998.1 to 90.81 | 0.1858 |
| Row 10 | -418 | -962.5 to 126.5 | 0.2863 |
| Row 11 | -441.4 | -985.9 to 103.0 | 0.2167 |
| Row 12 | -489.4 | -1034 to 55.12 | 0.1147 |

| **Test details** | **Predicted (LS) mean 1 (CON)** | **Predicted (LS) mean 2 (OE)** | **Predicted (LS) mean diff.** | **SE of diff.** | **N1** | **N2** | **t** | **DF** |
| --- | --- | --- | --- | --- | --- | --- | --- | --- |
| CON - OE |  |  |  |  |  |  |  |  |
| Row 1 | 13.98 | 10 | 3.979 | 188.8 | 11 | 12 | 0.021 | 252 |
| Row 2 | 71.67 | 156.2 | -84.54 | 188.8 | 11 | 12 | 0.448 | 252 |
| Row 3 | 364.4 | 410.6 | -46.24 | 188.8 | 11 | 12 | 0.245 | 252 |
| Row 4 | 682.4 | 701.8 | -19.4 | 188.8 | 11 | 12 | 0.103 | 252 |
| Row 5 | 922.8 | 1045 | -121.8 | 188.8 | 11 | 12 | 0.645 | 252 |
| Row 6 | 1099 | 1282 | -182.7 | 188.8 | 11 | 12 | 0.968 | 252 |
| Row 7 | 1250 | 1603 | -353.1 | 188.8 | 11 | 12 | 1.87 | 252 |
| Row 8 | 1587 | 2083 | -496.3 | 188.8 | 11 | 12 | 2.629 | 252 |
| Row 9 | 1664 | 2118 | -453.7 | 188.8 | 11 | 12 | 2.403 | 252 |
| Row 10 | 1678 | 2096 | -418 | 188.8 | 11 | 12 | 2.214 | 252 |
| Row 11 | 1674 | 2116 | -441.4 | 188.8 | 11 | 12 | 2.338 | 252 |
| Row 12 | 1596 | 2086 | -489.4 | 188.8 | 11 | 12 | 2.592 | 252 |

Extended Data Fig. 1L

|  | **Mean** | **SEM** | **N** |
| --- | --- | --- | --- |
| CON (TdTomato- AAV) | 1640 | 153.1 | 11 |
| OE (AAV-EfnB1) | 2100 | 129.9 | 12 |
| Statistics | t=2.303, df=21, p=0.0316 |  |  |

Extended Data Fig. 1N

|  | **CON (TdTomato-AAV)** | | | **OE (AAV-EfnB1)** | | |
| --- | --- | --- | --- | --- | --- | --- |
|  | **Mean** | **SEM** | **N** | **Mean** | **SEM** | **N** |
| Row 1 | 1148.089374 | 174.8662488 | 10 | 1698.71854 | 125.442 | 9 |
| Row 2 | 661.732774 | 107.1144174 | 10 | 1030.10501 | 60.8754 | 9 |
| Row 3 | 479.932174 | 87.00362781 | 10 | 684.246122 | 62.8916 | 9 |
| Row 4 | 436.256774 | 80.41994366 | 10 | 570.675011 | 70.5458 | 9 |
| Row 5 | 403.811974 | 73.08425131 | 10 | 533.2239 | 69.6977 | 9 |
| Row 6 | 354.457294 | 54.66534012 | 10 | 547.195011 | 78.1226 | 9 |
| Row 7 | 358.019174 | 70.67542126 | 10 | 516.339456 | 65.3455 | 9 |
| Row 8 | 348.358574 | 69.63018931 | 10 | 476.632789 | 64.9007 | 9 |
| Row 9 | 350.509385 | 68.57841112 | 10 | 467.692789 | 63.6222 | 9 |
| Row 10 | 332.484574 | 61.19191748 | 10 | 456.139456 | 66.8193 | 9 |

| **ANOVA table** | **SS** | **DF** | **MS** | **F (DFn, DFd)** | **P value** | **% of total variation** |
| --- | --- | --- | --- | --- | --- | --- |
| Stim x GENOTYPE | 848190 | 9 | 94243 | F (9, 153) = 4.761 | P<0.0001 | 2.678 |
| stim | 17574446 | 9 | 1952716 | F (9, 153) = 98.65 | P<0.0001 | 55.48 |
| GENOTYPE | 2103528 | 1 | 2103528 | F (1, 17) = 4.221 | P=0.0556 | 6.64 |
| cell | 8470934 | 17 | 498290 | F (17, 153) = 25.17 | P<0.0001 | 26.74 |

| **Šídák's multiple comparisons test** | **Predicted (LS) mean diff.** | **95.00% CI of**  **diff.** | **Adjusted P Value** |
| --- | --- | --- | --- |
| CON - OE |  |  |  |
| Row 1 | -550.6 | -889.6 to -211.7 | <0.0001 |
| Row 2 | -368.4 | -707.3 to -29.41 | 0.0237 |
| Row 3 | -204.3 | -543.3 to 134.6 | 0.6069 |
| Row 4 | -134.4 | -473.4 to 204.5 | 0.9522 |
| Row 5 | -129.4 | -468.4 to 209.5 | 0.9628 |
| Row 6 | -192.7 | -531.7 to 146.2 | 0.6834 |
| Row 7 | -158.3 | -497.3 to 180.6 | 0.8738 |
| Row 8 | -128.3 | -467.2 to 210.7 | 0.9649 |
| Row 9 | -117.2 | -456.1 to 221.8 | 0.9813 |
| Row 10 | -123.7 | -462.6 to 215.3 | 0.9726 |

| **Test details** | **Predicted (LS) mean (CON)** | **Predicted (LS) mean OE)** | **Predicted (LS) mean diff.** | **SE of diff.** | **N1** | **N2** | **t** | **DF** |
| --- | --- | --- | --- | --- | --- | --- | --- | --- |
| CON - OE |  |  |  |  |  |  |  |  |
| Row 1 | 1148 | 1699 | -550.6 | 119.5 | 10 | 9 | 4.608 | 170 |
| Row 2 | 661.7 | 1030 | -368.4 | 119.5 | 10 | 9 | 3.083 | 170 |
| Row 3 | 479.9 | 684.2 | -204.3 | 119.5 | 10 | 9 | 1.71 | 170 |
| Row 4 | 436.3 | 570.7 | -134.4 | 119.5 | 10 | 9 | 1.125 | 170 |
| Row 5 | 403.8 | 533.2 | -129.4 | 119.5 | 10 | 9 | 1.083 | 170 |
| Row 6 | 354.5 | 547.2 | -192.7 | 119.5 | 10 | 9 | 1.613 | 170 |
| Row 7 | 358 | 516.3 | -158.3 | 119.5 | 10 | 9 | 1.325 | 170 |
| Row 8 | 348.4 | 476.6 | -128.3 | 119.5 | 10 | 9 | 1.073 | 170 |
| Row 9 | 350.5 | 467.7 | -117.2 | 119.5 | 10 | 9 | 0.981 | 170 |
| Row 10 | 332.5 | 456.1 | -123.7 | 119.5 | 10 | 9 | 1.035 | 170 |

Extended Data Fig. 1 O

|  | **CON (TdTomato-AAV)** | | | **OE (AAV-EfnB1)** | | |
| --- | --- | --- | --- | --- | --- | --- |
|  | **Mean** | **SEM** | **N** | **Mean** | **SEM** | **N** |
| Row 1 | 0.601131398 | 0.043869929 | 10 | 0.619255 | 0.0385 | 9 |
| Row 2 | 0.441274881 | 0.048539836 | 10 | 0.410364 | 0.03564 | 9 |
| Row 3 | 0.400706696 | 0.05127993 | 10 | 0.338753 | 0.03676 | 9 |
| Row 4 | 0.383177088 | 0.063370978 | 10 | 0.319952 | 0.03771 | 9 |
| Row 5 | 0.342606784 | 0.046004388 | 10 | 0.322568 | 0.04039 | 9 |
| Row 6 | 0.330612747 | 0.039730343 | 10 | 0.304933 | 0.02996 | 9 |
| Row 7 | 0.325575805 | 0.047436177 | 10 | 0.282567 | 0.03502 | 9 |
| Row 8 | 0.319348201 | 0.035158411 | 10 | 0.28133 | 0.03484 | 9 |
| Row 9 | 0.308097903 | 0.038523211 | 10 | 0.27103 | 0.03495 | 9 |

Extended Data Fig. 1R

|  | **Mean** | **SEM** | **N** |
| --- | --- | --- | --- |
| CON | 1.000 | 0.0473 | 10 |
| OE (AAV-EfnB1) | 1.276 | 0.1089 | 10 |
| Statistics | t=2.322, df=18, p=0.0322 |  |  |

Extended Data Fig. 1S

|  | **Mean** | **SEM** | **N** |
| --- | --- | --- | --- |
| CON | 1.0000 | 0.0692 | 19 |
| OE (AAV-EfnB1) | 0.8009 | 0.0910 | 27 |
| Statistics | t=1.616, df=44, p=0.1132 |  |  |

Extended Data Fig. 1T

|  | **Mean** | **SEM** | **N** |
| --- | --- | --- | --- |
| CON | 1.000 | 0.0809 | 17 |
| OE (AAV-EfnB1) | 1.181 | 0.0946 | 17 |
| Statistics | t=1.460, df=32, p=0.1541 |  |  |
