# Supplementary material for: EphB2 Signaling Is Implicated in Astrocyte-Mediated Parvalbumin Inhibitory Synapse Development
Source: J Neurosci. 2024 Sep 26;44(45):e0154242024. doi: 10.1523/JNEUROSCI.0154-24.2024 (PMC11551896; doi:10.1523/JNEUROSCI.0154-24.2024)
Supplement: Table 2-1 — Statistical analysis for figure 2. Download Table 2-1, DOCX file. [file jneuro-44-e0154242024-s002.docx]

Extended Data Fig. 2D

|  | **Mean** | **SEM** | **N** |
| --- | --- | --- | --- |
| CON | 11.34 | 2.797 | 18 |
| KO | 4.577 | 0.9977 | 16 |
| Statistics | t=2.277, df=21.21, p=0.0332 |  |  |

Extended Data Fig. 2E

|  | **Mean** | **SEM** | **N** |
| --- | --- | --- | --- |
| CON | 1.781 | 0.4396 | 14 |
| KO | 0.6895 | 0.1839 | 13 |
| Statistics | t=2.290, df=17.37, p=0.0348 |  |  |

Extended Data Fig. 2F

|  | **Mean** | **SEM** | **N** |
| --- | --- | --- | --- |
| CON | 9.162 | 1.107 | 30 |
| KO | 3.909 | 0.5734 | 31 |
| Statistics | t=4.213, df=43.61, p=0.0001 |  |  |

Extended Data Fig. 2J

|  | **Mean** | **SEM** | **N** |
| --- | --- | --- | --- |
| CON | 1 | 0.1445 | 6 |
| KO | 0.4769 | 0.1142 | 6 |
| Statistics | t=2.841, df=10, p=0.0175 |  |  |

Extended Data Fig. 2K

|  | **Mean** | **SEM** | **N** |
| --- | --- | --- | --- |
| CON | 1 | 0.06623 | 23 |
| KO | 0.9022 | 0.04326 | 24 |
| Statistics | t=1.247, df=45, 0.2187 |  |  |
