# Supplementary material for: EphB2 Signaling Is Implicated in Astrocyte-Mediated Parvalbumin Inhibitory Synapse Development
Source: J Neurosci. 2024 Sep 26;44(45):e0154242024. doi: 10.1523/JNEUROSCI.0154-24.2024 (PMC11551896; doi:10.1523/JNEUROSCI.0154-24.2024)
Supplement: Table 3-1 — Statistical analysis for figure 3. Download Table 3-1, DOCX file. [file jneuro-44-e0154242024-s003.docx]

Extended Data Fig. 3B

|  | **Mean** | **SEM** | **N** |
| --- | --- | --- | --- |
| CON | 1468 | 218.6 | 8 |
| KO | 383.8 | 137 | 12 |
| Statistics | t=4.439, df=18, p=0.0003 |  |  |

Extended Data Fig. 3C

|  | **Mean** | **SEM** | **N** |
| --- | --- | --- | --- |
| CON | 7 | 3.591 | 8 |
| KO | 15.9 | 2.307 | 10 |
| Statistics | t=2.166, df=16, p=0.0458 |  |  |

Extended Data Fig. 3D

|  | **Mean** | **SEM** | **N** |
| --- | --- | --- | --- |
| CON | 2.375 | 0.625 | 8 |
| KO | 4.667 | 0.3333 | 12 |
| Statistics | MWU=17, DoF=18, p=0.0044 |  |  |

Extended Data Fig. 3E

**Stage 2**

|  | **Mean** | **SEM** | **N** |
| --- | --- | --- | --- |
| CON | 4.375 | 1.936 | 8 |
| KO | 5.000 | 1.291 | 10 |
| Statistics | MWU=32, DoF=16, p=0.4935 |  |  |

Extended Data Fig. 3F

**Stage 3**

|  | **Mean** | **SEM** | **N** |
| --- | --- | --- | --- |
| CON | 0.625 | 0.3239 | 8 |
| KO | 0.9 | 0.2769 | 10 |
| Statistics | MWU=32.5, DoF=16, p=0.5393 |  |  |

**Stage 4**

|  | **Mean** | **SEM** | **N** |
| --- | --- | --- | --- |
| CON | 0 | 0 | 8 |
| KO | 0.5 | 0.1667 | 10 |
| Statistics | MWU=20, DoF=16, p=0.0359 |  |  |

**Stage 5**

|  | **Mean** | **SEM** | **N** |
| --- | --- | --- | --- |
| CON | 0.25 | 0.1637 | 8 |
| KO | 0.9167 | 0.08333 | 12 |
| Statistics | MWU=16, DoF=18, p=0.0044 |  |  |

Extended Data Fig. 3G

|  | **Mean** | **SEM** | **N** |
| --- | --- | --- | --- |
| CON | 16 | 1.688 | 12 |
| KO | 18 | 1.628 | 12 |
| Statistics | MWU=63.50, p=0.6393 |  |  |

Extended Data Fig. 3H

|  | **Mean** | **SEM** | **N** |
| --- | --- | --- | --- |
| CON | 13.33 | 1.588 | 12 |
| KO | 9.5 | 1.091 | 12 |
| Statistics | MWU=44.50, p=0.1151 |  |  |

Extended Data Fig. 3I

|  | **Mean** | **SEM** | **N** |
| --- | --- | --- | --- |
| CON | 16.33 | 2.147 | 12 |
| KO | 14.08 | 1.406 | 12 |
| Statistics | MWU=57.50, p=0.4168 |  |  |

Extended Data Fig. 3J

|  | **Mean** | **SEM** | **N** |
| --- | --- | --- | --- |
| CON | 2.273 | 0.4491 | 11 |
| KO | 7.25 | 1.431 | 12 |
| Statistics | MWU=16.50, p=0.0013 |  |  |

Extended Data Fig. 3K

|  | **Mean** | **SEM** | **N** |
| --- | --- | --- | --- |
| CON | 4.833 | 1.347 | 12 |
| KO | 7.25 | 1.981 | 12 |
| Statistics | MWU=56, p=0.3666 |  |  |

Extended Data Fig 3L

|  | **Mean** | **SEM** | **N** |
| --- | --- | --- | --- |
| CON | 7 | 1.42 | 12 |
| KO | 14.06 | 0.8538 | 16 |
| Statistics | t=4.491, df=26 , p=0.0001 |  |  |

Extended Data Fig 3M

|  | **Mean** | **SEM** | **N** |
| --- | --- | --- | --- |
| CON | 0.6299 | 0.02448 | 11 |
| KO | 0.4693 | 0.04632 | 15 |
| Statistics | t=2.758, df=24, p=0.0110 |  |  |

Extended Data Fig 3N

|  | **Mean** | **SEM** | **N** |
| --- | --- | --- | --- |
| CON | 0.4837 | 0.03566 | 11 |
| KO | 0.4142 | 0.05942 | 16 |
| Statistics | t=0.8935, df=25, p=0.3801 |  |  |

Extended Data Fig 3O

| **ANOVA table** | **SS (Type III)** | **DF** | **MS** | **F (DFn, DFd)** | **P value** |
| --- | --- | --- | --- | --- | --- |
| Interaction | 975.4 | 1 | 975.4 | F (1, 50) = 4.279 | P=0.0438 |
| % Time | 3283 | 1 | 3283 | F (1, 50) = 14.40 | P=0.0004 |
| Genotype | 1901 | 1 | 1901 | F (1, 50) = 8.338 | P=0.0057 |
| Residual | 11399 | 50 | 228 |  |  |

| **Uncorrected Fisher's LSD** | **Predicted (LS) mean diff.** | **95.00% CI of diff.** | **Below threshold?** | **Summary** | **Individual P Value** |
| --- | --- | --- | --- | --- | --- |
|  |  |  |  |  |  |
| 1st 5 min:CON vs. 1st 5 min:KO | 3.426 | -8.453 to 15.30 | No | ns | 0.565 |
| 1st 5 min:CON vs. 2nd 5 min:CON | -24.52 | -37.45 to -11.59 | Yes | *** | 0.0004 |
| 1st 5 min:CON vs. 2nd 5 min:KO | -3.793 | -15.67 to 8.085 | No | ns | 0.5242 |
| 1st 5 min:KO vs. 2nd 5 min:CON | -27.94 | -39.82 to -16.07 | Yes | **** | <0.0001 |
| 1st 5 min:KO vs. 2nd 5 min:KO | -7.219 | -17.94 to 3.503 | No | ns | 0.1824 |
| 2nd 5 min:CON vs. 2nd 5 min:KO | 20.73 | 8.847 to 32.60 | Yes | *** | 0.001 |

| **Test details** | **Predicted (LS) mean 1** | **Predicted (LS) mean 2** | **Predicted (LS) mean diff.** | **SE of diff.** | **N1** | **N2** | **t** | **DF** |
| --- | --- | --- | --- | --- | --- | --- | --- | --- |
|  |  |  |  |  |  |  |  |  |
| 1st 5 min:CON vs. 1st 5 min:KO | 23.78 | 20.36 | 3.426 | 5.914 | 11 | 16 | 0.5792 | 50 |
| 1st 5 min:CON vs. 2nd 5 min:CON | 23.78 | 48.3 | -24.52 | 6.438 | 11 | 11 | 3.808 | 50 |
| 1st 5 min:CON vs. 2nd 5 min:KO | 23.78 | 27.58 | -3.793 | 5.914 | 11 | 16 | 0.6414 | 50 |
| 1st 5 min:KO vs. 2nd 5 min:CON | 20.36 | 48.3 | -27.94 | 5.914 | 16 | 11 | 4.725 | 50 |
| 1st 5 min:KO vs. 2nd 5 min:KO | 20.36 | 27.58 | -7.219 | 5.338 | 16 | 16 | 1.352 | 50 |
| 2nd 5 min:CON vs. 2nd 5 min:KO | 48.3 | 27.58 | 20.73 | 5.914 | 11 | 16 | 3.504 | 50 |

Extended Data Fig 3P

|  | **Mean** | **SEM** | **N** |
| --- | --- | --- | --- |
| CON | 12193 | 977.7 | 11 |
| KO | 12337 | 672.3 | 16 |
| Statistics | t=0.1259, df=25 , p=0.9008 |  |  |
