# Supplementary material for: EphB2 Signaling Is Implicated in Astrocyte-Mediated Parvalbumin Inhibitory Synapse Development
Source: J Neurosci. 2024 Sep 26;44(45):e0154242024. doi: 10.1523/JNEUROSCI.0154-24.2024 (PMC11551896; doi:10.1523/JNEUROSCI.0154-24.2024)
Supplement: Table 4-1 — Statistical analysis for figure 4. Download Table 4-1, DOCX file. [file jneuro-44-e0154242024-s004.docx]

Extended Data Fig. 4C

|  | **Mean** | **SEM** | **N** |
| --- | --- | --- | --- |
| CON (Contralateral side) | 1.0000 | 0.1750 | 8 |
| OE (AAV-EfnB1) | 0.4101 | 0.1529 | 7 |
| Statistics | t=2.503, df=13, p=0.0265 |  |  |

Extended Data Fig. 4D

|  | **Mean** | **SEM** | **N** |
| --- | --- | --- | --- |
| CON | 5.308 | 1.299 | 13 |
| OE (AAV-EfnB1) | 14.11 | 3.274 | 15 |
| Statistics | t=2.500, df=18.23, p=0.0222 |  |  |

Extended Data Fig. 4E

|  | **Mean** | **SEM** | **N** |
| --- | --- | --- | --- |
| CON (Contralateral side) | 71.77 | 10.15 | 8 |
| OE (AAV-EfnB1) | 56.41 | 14.10 | 7 |
| Statistics | t=0.9007, df=13, p=0.3841 |  |  |

Extended Data Fig. 4F

EphB on PV Soma

|  | **Mean** | **SEM** | **N** |
| --- | --- | --- | --- |
| CON (Contralateral side) | 34.55 | 3.252 | 17 |
| OE (AAV-EfnB1) | 33.02 | 2.754 | 22 |
| Statistics | t=0.3604, df=37, p=0.7206 |  |  |

EphB/Ephrin Colocalized on PV Soma

|  | **Mean** | **SEM** | **N** |
| --- | --- | --- | --- |
| CON (Contralateral side) | 11.74 | 2.385 | 25 |
| OE (AAV-EfnB1) | 13.01 | 1.897 | 22 |
| Statistics | t=0.4070, df=45, p=0.6859 |  |  |

Extended Data Fig. 4I

|  | **Mean** | **SEM** | **N** |
| --- | --- | --- | --- |
| CON | 1.0000 | 0.2089 | 14 |
| KO | 1.707 | 0.2353 | 11 |
| Statistics | t=2.246, df=23, p=0.0346 |  |  |

Extended Data Fig. 4J

|  | **Mean** | **SEM** | **N** |
| --- | --- | --- | --- |
| CON | 24.86 | 3.768 | 22 |
| KO | 10.11 | 2.076 | 13 |
| Statistics | t=3.427, df=30.73, p=0.0018 |  |  |

Extended Data Fig. 4K

|  | **Mean** | **SEM** | **N** |
| --- | --- | --- | --- |
| CON | 39.98 | 6.320 | 16 |
| KO | 34.40 | 4.913 | 11 |
| Statistics | t=0.6443, df=25, p=0.5252 |  |  |

Extended Data Fig. 4L

EphB on PV Soma

|  | **Mean** | **SEM** | **N** |
| --- | --- | --- | --- |
| CON | 17.73 | 2.404 | 25 |
| KO | 19.83 | 2.418 | 24 |
| Statistics | t=0.6153, df=47, p=0.5413 |  |  |

EphB/Ephrin Colocalized on PV Soma

|  | **Mean** | **SEM** | **N** |
| --- | --- | --- | --- |
| CON | 15.78 | 5.04 | 11 |
| KO | 21.66 | 6.203 | 10 |
| Statistics | t=1.607, df=47, p=0.1148 |  |  |
