# Supplementary material for: EphB2 Signaling Is Implicated in Astrocyte-Mediated Parvalbumin Inhibitory Synapse Development
Source: J Neurosci. 2024 Sep 26;44(45):e0154242024. doi: 10.1523/JNEUROSCI.0154-24.2024 (PMC11551896; doi:10.1523/JNEUROSCI.0154-24.2024)
Supplement: Table 5-1 — Statistical analysis for figure 5. Download Table 5-1, DOCX file. [file jneuro-44-e0154242024-s005.docx]

Extended Data Fig. 5C

|  | **Mean** | **SEM** | **N** |
| --- | --- | --- | --- |
| CON | 20.08 | 1.939 | 57 |
| KO | 11.09 | 1.337 | 50 |
| Statistics | t=3.817, df=96.88, p=0.0002 |  |  |

Extended Data Fig. 5E

|  | **Control** | | | **EphB2 KO** | | |
| --- | --- | --- | --- | --- | --- | --- |
|  | **Mean** | **SEM** | **N** | **Mean** | **SEM** | **N** |
| Row 1 | 2.102638889 | 2.102638889 | 18 | 3.12776528 | 1.8372 | 18 |
| Row 2 | 16.85020415 | 8.747717399 | 18 | 17.4042023 | 4.08248 | 18 |
| Row 3 | 59.02153842 | 17.68152908 | 18 | 115.053144 | 40.6332 | 18 |
| Row 4 | 207.9607731 | 49.60762179 | 18 | 381.894069 | 108.109 | 18 |
| Row 5 | 332.4908843 | 62.55374261 | 18 | 541.481458 | 112.982 | 18 |
| Row 6 | 449.5970046 | 69.49086545 | 18 | 572.572787 | 107.893 | 18 |
| Row 7 | 559.9054861 | 86.51236855 | 18 | 765.286343 | 102.174 | 18 |
| Row 8 | 1017.628333 | 144.5475011 | 18 | 1336.96148 | 152.244 | 18 |
| Row 9 | 1124.876898 | 140.2059316 | 18 | 1588.75412 | 148.413 | 18 |
| Row 10 | 1150.944676 | 150.2438929 | 18 | 1666.01607 | 151.082 | 18 |
| Row 11 | 1188.762732 | 148.0025367 | 18 | 1626.41806 | 154.621 | 18 |
| Row 12 | 1178.865 | 153.4392681 | 18 | 1623.74032 | 152.635 | 18 |

| **ANOVA table** | **SS** | **DF** | **MS** | **F (DFn, DFd)** | **P value** | **% of total variation** |
| --- | --- | --- | --- | --- | --- | --- |
| led power x genotype | 3431007 | 11 | 311910 | F (11, 374) = 2.946 | P=0.0009 | 1.468 |
| led power | 134689336 | 11 | 1.2E+07 | F (11, 374) = 115.7 | P<0.0001 | 57.63 |
| genotype | 6525564 | 1 | 6525564 | F (1, 34) = 4.484 | P=0.0416 | 2.792 |
| cell | 49479414 | 34 | 1455277 | F (34, 374) = 13.75 | P<0.0001 | 21.17 |

| **Šídák's multiple comparisons test** | **Mean Diff.** | **95.00% CI of diff.** | **Adjusted P Value** |
| --- | --- | --- | --- |
| Control - EphB2 KO |  |  |  |
| Row 1 | -1.025 | -448.6 to 446.6 | >0.9999 |
| Row 2 | -0.554 | -448.2 to 447.1 | >0.9999 |
| Row 3 | -56.03 | -503.7 to 391.6 | >0.9999 |
| Row 4 | -173.9 | -621.6 to 273.7 | 0.975 |
| Row 5 | -209 | -656.6 to 238.6 | 0.9081 |
| Row 6 | -123 | -570.6 to 324.6 | 0.9988 |
| Row 7 | -205.4 | -653.0 to 242.2 | 0.9179 |
| Row 8 | -319.3 | -767.0 to 128.3 | 0.3947 |
| Row 9 | -463.9 | -911.5 to -16.26 | 0.0362 |
| Row 10 | -515.1 | -962.7 to -67.45 | 0.0122 |
| Row 11 | -437.7 | -885.3 to 9.965 | 0.0606 |
| Row 12 | -444.9 | -892.5 to 2.745 | 0.0527 |

| **Test details** | **Mean 1** | **Mean 2** | **Mean Diff.** | **SE of diff.** | **N1** | **N2** | **t** | **DF** |
| --- | --- | --- | --- | --- | --- | --- | --- | --- |
| Control - EphB2 KO |  |  |  |  |  |  |  |  |
| Row 1 | 2.103 | 3.128 | -1.025 | 155.7 | 18 | 18 | 0.007 | 408 |
| Row 2 | 16.85 | 17.4 | -0.554 | 155.7 | 18 | 18 | 0.004 | 408 |
| Row 3 | 59.02 | 115.1 | -56.03 | 155.7 | 18 | 18 | 0.36 | 408 |
| Row 4 | 208 | 381.9 | -173.9 | 155.7 | 18 | 18 | 1.117 | 408 |
| Row 5 | 332.5 | 541.5 | -209 | 155.7 | 18 | 18 | 1.342 | 408 |
| Row 6 | 449.6 | 572.6 | -123 | 155.7 | 18 | 18 | 0.79 | 408 |
| Row 7 | 559.9 | 765.3 | -205.4 | 155.7 | 18 | 18 | 1.319 | 408 |
| Row 8 | 1018 | 1337 | -319.3 | 155.7 | 18 | 18 | 2.05 | 408 |
| Row 9 | 1125 | 1589 | -463.9 | 155.7 | 18 | 18 | 2.978 | 408 |
| Row 10 | 1151 | 1666 | -515.1 | 155.7 | 18 | 18 | 3.307 | 408 |
| Row 11 | 1189 | 1626 | -437.7 | 155.7 | 18 | 18 | 2.81 | 408 |
| Row 12 | 1179 | 1624 | -444.9 | 155.7 | 18 | 18 | 2.856 | 408 |

Extended Data Fig. 5F

|  | **Mean** | **SEM** | **N** |
| --- | --- | --- | --- |
| CON | 1243 | 152.2 | 18 |
| KO | 1713 | 151.9 | 18 |
| Statistics | t=2.188, df=34, p=0.0357 |  |  |

Extended Data Fig. 5H

|  | **Control** | | | **EphB2 KO** | | |
| --- | --- | --- | --- | --- | --- | --- |
|  | **Mean** | **SEM** | **N** | **Mean** | **SEM** | **N** |
| Row 1 | 880.8733333 | 134.6552327 | 18 | 1186.33333 | 128.857 | 18 |
| Row 2 | 475.2516667 | 81.07735741 | 18 | 657.527778 | 86.1582 | 18 |
| Row 3 | 319.9244444 | 73.53078369 | 18 | 395.863889 | 63.0547 | 18 |
| Row 4 | 221.3628333 | 55.65583216 | 18 | 308.22 | 47.4455 | 18 |
| Row 5 | 184.3416111 | 53.43840089 | 18 | 319.672778 | 52.457 | 18 |
| Row 6 | 165.7156111 | 44.57906141 | 18 | 240.212778 | 35.5261 | 18 |
| Row 7 | 158.3108889 | 46.47876765 | 18 | 225.508333 | 42.2781 | 18 |
| Row 8 | 191.9483333 | 51.68501854 | 18 | 204.639444 | 31.1381 | 18 |
| Row 9 | 164.4002222 | 46.67343381 | 18 | 212.595 | 36.1207 | 18 |
| Row 10 | 152.2038889 | 45.83747106 | 18 | 170.752056 | 30.1102 | 18 |

| **ANOVA table** | **SS** | **DF** | **MS** | **F (DFn, DFd)** | **P value** | **% of total**  **variation** |
| --- | --- | --- | --- | --- | --- | --- |
| STIMULUS # x GENOTYPE | 626811 | 9 | 69646 | F (9, 306) = 2.473 | P=0.0098 | 1.21 |
| STIMULUS # | 23818992 | 9 | 2646555 | F (9, 306) = 93.98 | P<0.0001 | 45.99 |
| GENOTYPE | 912631 | 1 | 912631 | F (1, 34) = 1.741 | P=0.1958 | 1.762 |
| CELL | 17821268 | 34 | 524155 | F (34, 306) = 18.61 | P<0.0001 | 34.41 |

| **Šídák's multiple comparisons test** | **Mean Diff.** | **95.00% CI of**  **diff.** | **Adjusted P Value** |
| --- | --- | --- | --- |
| Control - EphB2 KO |  |  |  |
| Row 1 | -305.5 | -567.4 to -43.53 | 0.0112 |
| Row 2 | -182.3 | -444.2 to 79.66 | 0.4056 |
| Row 3 | -75.94 | -337.9 to 186.0 | 0.9953 |
| Row 4 | -86.86 | -348.8 to 175.1 | 0.9867 |
| Row 5 | -135.3 | -397.3 to 126.6 | 0.7945 |
| Row 6 | -74.5 | -336.4 to 187.4 | 0.9959 |
| Row 7 | -67.2 | -329.1 to 194.7 | 0.9983 |
| Row 8 | -12.69 | -274.6 to 249.2 | >0.9999 |
| Row 9 | -48.19 | -310.1 to 213.7 | >0.9999 |
| Row 10 | -18.55 | -280.5 to 243.4 | >0.9999 |

Extended Data Fig. 5I

|  | **Control** | | | **EphB2 KO** | | |
| --- | --- | --- | --- | --- | --- | --- |
|  | **Mean** | **SEM** | **N** | **Mean** | **SEM** | **N** |
| Row 1 | 0.54707378 | 0.051463777 | 18 | 0.54783267 | 0.02833 | 18 |
| Row 2 | 0.333758276 | 0.042720405 | 18 | 0.32195526 | 0.02679 | 18 |
| Row 3 | 0.223934361 | 0.034679769 | 18 | 0.25227514 | 0.02166 | 18 |
| Row 4 | 0.176959499 | 0.035315223 | 18 | 0.26132029 | 0.0232 | 18 |
| Row 5 | 0.165694848 | 0.028940159 | 18 | 0.20095088 | 0.0191 | 18 |
| Row 6 | 0.153018146 | 0.029826447 | 18 | 0.18557535 | 0.01941 | 18 |
| Row 7 | 0.159726252 | 0.032081979 | 18 | 0.17583559 | 0.01732 | 18 |
| Row 8 | 0.133355156 | 0.03157291 | 18 | 0.18120978 | 0.02177 | 18 |
| Row 9 | 0.1511695 | 0.029764799 | 18 | 0.13852005 | 0.0165 | 18 |

| **ANOVA table** | **SS** | **DF** | **MS** | **F (DFn, DFd)** | **P value** | **% of total variation** |
| --- | --- | --- | --- | --- | --- | --- |
| STIMULUS # x GENOTYPE | 0.06891 | 8 | 0.00861 | F (8, 272) = 1.767 | P=0.0836 | 0.7083 |
| STIMULUS # | 4.752 | 8 | 0.594 | F (8, 272) = 121.8 | P<0.0001 | 48.85 |
| GENOTYPE | 0.04875 | 1 | 0.04875 | F (1, 34) = 0.4691 | P=0.4980 | 0.501 |
| CELL | 3.533 | 34 | 0.1039 | F (34, 272) = 21.31 | P<0.0001 | 36.31 |

| **Šídák's multiple comparisons test** | **Mean Diff.** | **95.00% CI of diff.** | **Adjusted P Value** |
| --- | --- | --- | --- |
| Control - EphB2 KO |  |  |  |
| Row 1 | -0.0007589 | -0.1177 to 0.1162 | >0.9999 |
| Row 2 | 0.0118 | -0.1052 to 0.1288 | >0.9999 |
| Row 3 | -0.02834 | -0.1453 to 0.08865 | 0.9981 |
| Row 4 | -0.08436 | -0.2013 to 0.03263 | 0.3422 |
| Row 5 | -0.03526 | -0.1522 to 0.08173 | 0.9902 |
| Row 6 | -0.03256 | -0.1495 to 0.08443 | 0.9945 |
| Row 7 | -0.01611 | -0.1331 to 0.1009 | >0.9999 |
| Row 8 | -0.04785 | -0.1648 to 0.06913 | 0.9297 |
| Row 9 | 0.01265 | -0.1043 to 0.1296 | >0.9999 |

| **Test details** | **Mean 1** | **Mean 2** | **Mean Diff.** | **SE of diff.** | **N1** | **N2** | **t** | **DF** |
| --- | --- | --- | --- | --- | --- | --- | --- | --- |
| Control - EphB2 KO |  |  |  |  |  |  |  |  |
| Row 1 | 0.5471 | 0.5478 | - 0.00076 | 0.042 | 18 | 18 | 0.018 | 306 |
| Row 2 | 0.3338 | 0.322 | 0.0118 | 0.042 | 18 | 18 | 0.281 | 306 |
| Row 3 | 0.2239 | 0.2523 | - 0.02834 | 0.042 | 18 | 18 | 0.675 | 306 |
| Row 4 | 0.177 | 0.2613 | - 0.08436 | 0.042 | 18 | 18 | 2.008 | 306 |
| Row 5 | 0.1657 | 0.201 | - 0.03526 | 0.042 | 18 | 18 | 0.839 | 306 |
| Row 6 | 0.153 | 0.1856 | - 0.03256 | 0.042 | 18 | 18 | 0.775 | 306 |
| Row 7 | 0.1597 | 0.1758 | - 0.01611 | 0.042 | 18 | 18 | 0.384 | 306 |
| Row 8 | 0.1334 | 0.1812 | - 0.04785 | 0.042 | 18 | 18 | 1.139 | 306 |
| Row 9 | 0.1512 | 0.1385 | 0.01265 | 0.042 | 18 | 18 | 0.301 | 306 |

| **Test details** | **Mean 1** | **Mean 2** | **Mean Diff.** | **SE of diff.** | **N1** | **N2** | **t** | **DF** |
| --- | --- | --- | --- | --- | --- | --- | --- | --- |
| Control - EphB2 KO |  |  |  |  |  |  |  |  |
| Row 1 | 880.9 | 1186 | -305.5 | 92.95 | 18 | 18 | 3.286 | 340 |
| Row 2 | 475.3 | 657.5 | -182.3 | 92.95 | 18 | 18 | 1.961 | 340 |
| Row 3 | 319.9 | 395.9 | -75.94 | 92.95 | 18 | 18 | 0.817 | 340 |
| Row 4 | 221.4 | 308.2 | -86.86 | 92.95 | 18 | 18 | 0.934 | 340 |
| Row 5 | 184.3 | 319.7 | -135.3 | 92.95 | 18 | 18 | 1.456 | 340 |
| Row 6 | 165.7 | 240.2 | -74.5 | 92.95 | 18 | 18 | 0.802 | 340 |
| Row 7 | 158.3 | 225.5 | -67.2 | 92.95 | 18 | 18 | 0.723 | 340 |
| Row 8 | 191.9 | 204.6 | -12.69 | 92.95 | 18 | 18 | 0.137 | 340 |
| Row 9 | 164.4 | 212.6 | -48.19 | 92.95 | 18 | 18 | 0.519 | 340 |
| Row 10 | 152.2 | 170.8 | -18.55 | 92.95 | 18 | 18 | 0.2 | 340 |

Extended Data Fig 5J

|  | **Mean** | **SEM** | **N** |
| --- | --- | --- | --- |
| CON | 1340 | 196.9 | 10 |
| KO | 1800 | 0 | 9 |
| Statistics | t=2.208, df=17, p=0.0412 |  |  |

Extended Data Fig 5K

|  | **Mean** | **SEM** | **N** |
| --- | --- | --- | --- |
| CON | 45.2 | 28.37 | 10 |
| KO | 0 | 0 | 9 |
| Statistics | t=1.507, df=17, p=0.1502 |  |  |

Extended Data Fig 5L

| P value and statistical significance |  |
| --- | --- |
| Test | Fisher's exact test |
| P value | 0.3034 |
| P value summary | ns |
| One- or two-sided | Two-sided |
| Statistically significant (P < 0.05)? | No |

| **Effect size** | **Value** | **95% CI** |
| --- | --- | --- |
| Odds ratio | 0.1667 | 0.01248 to 1.699 |
| Reciprocal of odds ratio | 6 | 0.5886 to 80.14 |

| **Methods used to compute CIs** |  |
| --- | --- |
| Odds ratio | Baptista-Pike |

Extended Data Fig 5M

| **ANOVA table** | **SS (Type III)** | **DF** | **MS** | **F (DFn, DFd)** | **P value** |
| --- | --- | --- | --- | --- | --- |
| Interaction | 133.5 | 1 | 133.5 | F (1, 40) = 1.556 | P=0.2195 |
| Row Factor | 1472 | 1 | 1472 | F (1, 40) = 17.16 | P=0.0002 |
| Column Factor | 552 | 1 | 552 | F (1, 40) = 6.436 | P=0.0152 |
| Residual | 3431 | 40 | 85.78 |  |  |

| **Tukey's multiple comparisons test** | **Predicted (LS) mean diff.** | **95.00% CI of diff.** | **Below threshold?** | **Summary** | **Adjusted P Value** |
| --- | --- | --- | --- | --- | --- |
|  |  |  |  |  |  |
| 1st 5 min:CON vs. 1st 5 min:KO | -10.75 | -21.51 to 0.01795 | No | ns | 0.0505 |
| 1st 5 min:CON vs. 2nd 5 min:CON | -15.31 | -25.04 to -5.570 | Yes | *** | 0.0008 |
| 1st 5 min:CON vs. 2nd 5 min:KO | -18.97 | -29.73 to -8.204 | Yes | *** | 0.0002 |
| 1st 5 min:KO vs. 2nd 5 min:CON | -4.561 | -15.33 to 6.204 | No | ns | 0.67 |
| 1st 5 min:KO vs. 2nd 5 min:KO | -8.222 | -19.92 to 3.481 | No | ns | 0.2514 |
| 2nd 5 min:CON vs. 2nd 5 min:KO | -3.662 | -14.43 to 7.103 | No | ns | 0.7987 |

| **Test details** | **Predicted (LS) mean 1** | **Predicted (LS) mean 2** | **Predicted (LS) mean diff.** | **SE of diff.** | **N1** | **N2** | **q** | **DF** |
| --- | --- | --- | --- | --- | --- | --- | --- | --- |
|  |  |  |  |  |  |  |  |  |
| 1st 5 min:CON vs. 1st 5 min:KO | 18.23 | 28.98 | -10.75 | 4.016 | 13 | 9 | 3.784 | 40 |
| 1st 5 min:CON vs. 2nd 5 min:CON | 18.23 | 33.54 | -15.31 | 3.633 | 13 | 13 | 5.959 | 40 |
| 1st 5 min:CON vs. 2nd 5 min:KO | 18.23 | 37.2 | -18.97 | 4.016 | 13 | 9 | 6.68 | 40 |
| 1st 5 min:KO vs. 2nd 5 min:CON | 28.98 | 33.54 | -4.561 | 4.016 | 9 | 13 | 1.606 | 40 |
| 1st 5 min:KO vs. 2nd 5 min:KO | 28.98 | 37.2 | -8.222 | 4.366 | 9 | 9 | 2.663 | 40 |
| 2nd 5 min:CON vs. 2nd 5 min:KO | 33.54 | 37.2 | -3.662 | 4.016 | 13 | 9 | 1.289 | 40 |

Extended Data Fig 5N

|  | **Mean** | **SEM** | **N** |
| --- | --- | --- | --- |
| CON | 13837 | 467.1 | 13 |
| KO | 16199 | 570.2 | 9 |
| Statistics | t=3.213, df=20, p=0.0044 |  |  |

Extended Data Fig 5O

|  | **Mean** | **SEM** | **N** |
| --- | --- | --- | --- |
| CON | 10.69 | 1.696 | 13 |
| KO | 9.444 | 1.659 | 9 |
| Statistics | t=0.5060, df=20, p=0.6184 |  |  |
