# Supplementary material for: EphB2 Signaling Is Implicated in Astrocyte-Mediated Parvalbumin Inhibitory Synapse Development
Source: J Neurosci. 2024 Sep 26;44(45):e0154242024. doi: 10.1523/JNEUROSCI.0154-24.2024 (PMC11551896; doi:10.1523/JNEUROSCI.0154-24.2024)
Supplement: Table 6-1 — Statistical analysis for figure 6. Download Table 6-1, DOCX file. [file jneuro-44-e0154242024-s006.docx]

Extended Data Fig. 6C

|  | **Mean** | **SEM** | **N** |
| --- | --- | --- | --- |
| CON | 1 | 0.08391 | 46 |
| HET | 1.292 | 0.2241 | 19 |
| KO | 1.295 | 0.08499 | 22 |

| **Brown-Forsythe ANOVA test** |  |
| --- | --- |
| F* (DFn, DFd) | 1.837 (2.000,  32.24) |
| P value | 0.1756 |

| **Welch's ANOVA test** |  |
| --- | --- |
| W (DFn, DFd) | 3.193 (2.000,  38.96) |
| P value | 0.052 |

| **Dunnett's T3 multiple**  **comparisons test** | **Mean Diff.** | **95.00% CI of diff.** | **Adjusted P Value** |  |  |  |  |  |
| --- | --- | --- | --- | --- | --- | --- | --- | --- |
| CON vs. HET | -0.2921 | -0.9061 to 0.3218 | 0.5407 |  |  |  |  |  |
| CON vs. KO | -0.2949 | -0.5883 to -0.001403 | 0.0486 |  |  |  |  |  |
| HET vs. KO | -0.002742 | -0.6177 to 0.6122 | >0.9999 |  |  |  |  |  |
| **Test details** | **Mean 1** | **Mean 2** | **Mean Diff.** | **SE of diff.** | **n1** | **n2** | **t** | **DF** |
| CON vs. HET | 1 | 1.292 | -0.2921 | 0.2393 | 46 | 19 | 1.221 | 23.22 |
| CON vs. KO | 1 | 1.295 | -0.2949 | 0.1194 | 46 | 22 | 2.469 | 56.74 |
| HET vs. KO | 1.292 | 1.295 | - 0.00274 | 0.2397 | 19 | 22 | 0.011 | 23.14 |

Extended Data Fig. 6D

|  | **Mean** | **SEM** | **N** |
| --- | --- | --- | --- |
| CON | 1 | 0.03983 | 165 |
| HET | 1.287 | 0.06783 | 72 |
| KO | 1.459 | 0.09471 | 97 |

| **Brown-Forsythe ANOVA test** |  |
| --- | --- |
| F* (DFn, DFd) | 13.50 (2.000,  202.7) |
| P value | <0.0001 |

Extended Data Fig. 6E

|  | **Mean** | **SEM** | **N** |
| --- | --- | --- | --- |
| CON | 26.70 | 0.6778 | 5 |
| KO | 24.94 | 1.783 | 5 |
| Statistics | t=0.9220, df=8, p=0.3835 |  |  |

Extended Data Fig. 6I

|  | **Mean** | **SEM** | **N** |
| --- | --- | --- | --- |
| CON | 3.178 | 0.1490 | 23 |
| KO | 2.882 | 0.1404 | 23 |
| Statistics | t=1.447, df=44, p=0.1551 | | |

Extended Data Fig. 6J

|  | **Mean** | **SEM** | **N** |
| --- | --- | --- | --- |
| CON | 2.014 | 0.1326 | 23 |
| KO | 2.828 | 0.2624 | 23 |
| Statistics | t=2.767, df=32.55, p=0.0092 | | |

Extended Data Fig. 6K

|  | **Mean** | **SEM** | **N** |
| --- | --- | --- | --- |
| CON | 2.460 | 0.2152 | 23 |
| KO | 3.324 | 0.2640 | 23 |
| Statistics | t=2.539, df=44, p=0.0147 | | |

Extended Data Fig. 6L

|  | **Mean** | **SEM** | **N** |
| --- | --- | --- | --- |
| CON | 1.948 | 0.1896 | 23 |
| KO | 2.104 | 0.1250 | 23 |
| Statistics | t=0.6854, df=44, p=0.4967 | | |

Extended Data Fig. 6M

|  | **Mean** | **SEM** | **N** |
| --- | --- | --- | --- |
| CON | 0.3648 | 0.01664 | 23 |
| KO | 0.6017 | 0.04425 | 23 |
| Statistics | t=5.012, df=44, p<0.0001 | | |

Extended Data Fig. 6N

| ANOVA Summary | |
| --- | --- |
| F | 52.18 |
| P-value | <0.0001 |
| R-Squared | 0.9383 |

| **ANOVA table** | SS | DF | MS | F (DFn, DFd) | P value |
| --- | --- | --- | --- | --- | --- |
| Treatment (between columns) | 9244 | 7 | 1321 | F (7, 24) = 52.18 | P<0.0001 |
| Residual (within columns) | 607.4 | 24 | 25.31 |  |  |
| Total | 9852 | 31 |  |  |  |

| **Šídák's multiple comparisons test** | Mean Diff. | 95.00% CI of diff. | Adjusted P Value |
| --- | --- | --- | --- |
| CON 0-0.25 vs. KO 0-0.25 | 21.66 | 12.09 to 31.24 | <0.0001 |
| CON 0.25-0.5 vs. KO 0.25-0.5 | 16.64 | 7.069 to 26.22 | 0.0004 |
| CON 0.5-1 vs. KO 0.5-1 | -29.03 | -38.61 to -19.45 | <0.0001 |
| CON >1 vs. KO >1 | -9.280 | -18.86 to 0.2955 | 0.0602 |

| **Test details** | Mean 1 | Mean 2 | Mean Diff. | SE of diff. | n1 | n2 | t | DF |
| --- | --- | --- | --- | --- | --- | --- | --- | --- |
| CON 0-0.25 vs. KO 0-0.25 | 31.65 | 9.984 | 21.66 | 3.557 | 4 | 4 | 6.090 | 24 |
| CON 0.25-0.5 vs. KO 0.25-0.5 | 50.19 | 33.55 | 16.64 | 3.557 | 4 | 4 | 4.679 | 24 |
| CON 0.5-1 vs. KO 0.5-1 | 17.45 | 46.48 | -29.03 | 3.557 | 4 | 4 | 8.161 | 24 |
| CON >1 vs. KO >1 | 0.7092 | 9.989 | -9.280 | 3.557 | 4 | 4 | 2.609 | 24 |

| **Welch's ANOVA test** |  |
| --- | --- |
| W (DFn, DFd) | 13.91 (2.000,  154.3) |
| P value | <0.0001 |

| **Dunnett's T3 multiple comparisons test** | **Mean Diff.** | **95.00% CI**  **of diff.** |  | **Adjusted P Value** |
| --- | --- | --- | --- | --- |
| CON vs. HET | -0.2872 | -0.4775 to -  0.09684 |  | 0.0012 |
| CON vs. KO | -0.4586 | -0.7070 to -  0.2102 |  | <0.0001 |
| HET vs. KO | -0.1714 | -0.4524 to  0.1095 |  | 0.3695 |

| **Test details** | **Mean 1** | **Mean 2** | **Mean Diff.** | **SE of diff.** | **n1** | **n2** | **t** | **DF** |
| --- | --- | --- | --- | --- | --- | --- | --- | --- |
| CON vs. HET | 1 | 1.287 | -0.2872 | 0.07866 | 165 | 72 | 3.651 | 122.1 |
| CON vs. KO | 1 | 1.459 | -0.4586 | 0.1027 | 165 | 97 | 4.464 | 130.6 |
| HET vs. KO | 1.287 | 1.459 | -0.1714 | 0.1165 | 72 | 97 | 1.472 | 162.1 |

Extended Data Fig. 6O

|  | **Mean** | **SEM** | **N** |
| --- | --- | --- | --- |
| CON | 0.07391 | 0.009191 | 23 |
| KO | 0.2665 | 0.03569 | 23 |
| Statistics | t=5.226, df=24.91, p<0.0001 | | |

Extended Data Fig. 6P

|  | **Mean** | **SEM** | **N** |
| --- | --- | --- | --- |
| CON | 0.1304 | 0.02108 | 23 |
| KO | 0.6104 | 0.09946 | 23 |
| Statistics | t=4.721, df=23.97, p<0.0001 | | |
