# Supplementary material for: EphB2 Signaling Is Implicated in Astrocyte-Mediated Parvalbumin Inhibitory Synapse Development
Source: J Neurosci. 2024 Sep 26;44(45):e0154242024. doi: 10.1523/JNEUROSCI.0154-24.2024 (PMC11551896; doi:10.1523/JNEUROSCI.0154-24.2024)
Supplement: Table 7-1 — Statistical analysis for figure 7. Download Table 7-1, DOCX file. [file jneuro-44-e0154242024-s007.docx]

Extended Data Fig. 7B

|  | **EphB2 KO +AAV-Efnb1** | | | **EphB2 KO +AAV-TdTomato** | | |
| --- | --- | --- | --- | --- | --- | --- |
|  | **Mean** | **SEM** | **N** | **Mean** | **SEM** | **N** |
| Row 1 | 9.581777781 | 5.460156345 | 12 | 2.72353333 | 1.81685 | 10 |
| Row 2 | 73.44755558 | 35.96171683 | 12 | 15.2458 | 3.5562 | 10 |
| Row 3 | 424.6563125 | 111.8066813 | 12 | 194.646583 | 61.3969 | 10 |
| Row 4 | 899.1635975 | 174.0896373 | 12 | 642.5778 | 202.077 | 10 |
| Row 5 | 1214.541472 | 189.736623 | 12 | 899.756658 | 211.049 | 10 |
| Row 6 | 1396.674472 | 185.6184028 | 12 | 1160.99148 | 222.14 | 10 |
| Row 7 | 1624.684861 | 187.0770618 | 12 | 1368.11588 | 250.476 | 10 |
| Row 8 | 2151.947918 | 188.3888436 | 12 | 1759.21698 | 233.181 | 10 |
| Row 9 | 2302.915973 | 195.0019804 | 12 | 2000.69983 | 212.353 | 10 |
| Row 10 | 2331.35764 | 191.9217867 | 12 | 2060.73083 | 224.828 | 10 |
| Row 11 | 2288.072222 | 194.3589019 | 12 | 2058.09667 | 227.99 | 10 |
| Row 12 | 2264.691665 | 189.1553335 | 12 | 1996.2725 | 229.127 | 10 |

| **ANOVA table** | **SS** | **DF** | **MS** | **F (DFn, DFd)** | **P value** | **% of total variation** |
| --- | --- | --- | --- | --- | --- | --- |
| LED power x genotype | 667834 | 11 | 60712 | F (11, 220) = 0.3973 | P=0.9561 | 0.25 |
| LED power | 174973883 | 11 | 1.6E+07 | F (2.460, 49.19) = 104.1 | P<0.0001 | 65.49 |
| genotype | 3621552 | 1 | 3621552 | F (1, 20) = 1.409 | P=0.2492 | 1.355 |
| cell | 51422141 | 20 | 2571107 | F (20, 220) = 16.83 | P<0.0001 | 19.25 |

| **Šídák's multiple comparisons test** | **Mean Diff.** | | **95.00% CI**  **of diff.** | **Adjusted P Value** |
| --- | --- | --- | --- | --- |
| EphB2 KO +AAV-Efnb1 - EphB2 KO +AAV-TdTomato | |  |  |  |
| Row 1 | 6.858 | | -12.91 to  26.63 | 0.9703 |
| Row 2 | 58.2 | | -70.82 to  187.2 | 0.8246 |
| Row 3 | 230 | | -191.3 to  651.3 | 0.6748 |
| Row 4 | 256.6 | | -609.9 to  1123 | 0.9941 |
| Row 5 | 314.8 | | -604.9 to  1234 | 0.9809 |
| Row 6 | 235.7 | | -706.6 to  1178 | 0.9987 |
| Row 7 | 256.6 | | -770.3 to  1283 | 0.9986 |
| Row 8 | 392.7 | | -585.4 to  1371 | 0.9376 |
| Row 9 | 302.2 | | -631.1 to  1236 | 0.9878 |
| Row 10 | 270.6 | | -690.2 to  1231 | 0.9962 |
| Row 11 | 230 | | -743.9 to  1204 | 0.9993 |
| Row 12 | 268.4 | | -699.5 to  1236 | 0.9966 |

| **Test details** | **Mean 1** | **Mean 2** | **Mean Diff.** | **SE of diff.** | **N1** | **N2** | **t** | **DF** |
| --- | --- | --- | --- | --- | --- | --- | --- | --- |
| EphB2 KO +AAV-  Efnb1 - EphB2 KO  +AAV-TdTomato |  |  |  |  |  |  |  |  |
| Row 1 | 9.582 | 2.724 | 6.858 | 5.754 | 12 | 10 | 1.192 | 13.37 |
| Row 2 | 73.45 | 15.25 | 58.2 | 36.14 | 12 | 10 | 1.611 | 11.21 |
| Row 3 | 424.7 | 194.6 | 230 | 127.6 | 12 | 10 | 1.803 | 16.77 |
| Row 4 | 899.2 | 642.6 | 256.6 | 266.7 | 12 | 10 | 0.962 | 18.83 |
| Row 5 | 1215 | 899.8 | 314.8 | 283.8 | 12 | 10 | 1.109 | 19.18 |
| Row 6 | 1397 | 1161 | 235.7 | 289.5 | 12 | 10 | 0.814 | 18.55 |
| Row 7 | 1625 | 1368 | 256.6 | 312.6 | 12 | 10 | 0.821 | 17.41 |
| Row 8 | 2152 | 1759 | 392.7 | 299.8 | 12 | 10 | 1.31 | 18.23 |
| Row 9 | 2303 | 2001 | 302.2 | 288.3 | 12 | 10 | 1.048 | 19.33 |
| Row 10 | 2331 | 2061 | 270.6 | 295.6 | 12 | 10 | 0.916 | 18.75 |
| Row 11 | 2288 | 2058 | 230 | 299.6 | 12 | 10 | 0.768 | 18.74 |
| Row 12 | 2265 | 1996 | 268.4 | 297.1 | 12 | 10 | 0.903 | 18.44 |

Extended Data Fig. 7C

|  | **Mean** | **SEM** | **N** |
| --- | --- | --- | --- |
| EphB2 KO +AAV-  Efnb1 | 2368 | 188.5 | 12 |
| EphB2 KO +AAV-  TdTomato | 2137 | 229.6 | 10 |
| Statistics | t=0.7851, df=20, p=0.4416 |  |  |

Extended Data Fig. 7E

|  | **EphB2 KO +AAV-Efnb1** | | | **EphB2 KO +AAV-TdTomato** | | |
| --- | --- | --- | --- | --- | --- | --- |
|  | **Mean** | **SEM** | **N** | **Mean** | **SEM** | **N** |
| Row 1 | 1852.32 | 249.125056 | 10 | 1593.68889 | 199.538 | 9 |
| Row 2 | 1355.39 | 212.3548066 | 10 | 926.888889 | 197.644 | 9 |
| Row 3 | 983.43 | 179.0582717 | 10 | 617.9 | 154.727 | 9 |
| Row 4 | 869.363 | 153.7396536 | 10 | 518.322222 | 137.531 | 9 |
| Row 5 | 775.426 | 138.4154568 | 10 | 470.766667 | 127.071 | 9 |
| Row 6 | 685.907 | 126.0691255 | 10 | 465.811111 | 125.2 | 9 |
| Row 7 | 660.962 | 119.6477843 | 10 | 432.733333 | 116.976 | 9 |
| Row 8 | 696.102 | 136.8061477 | 10 | 428.122222 | 114.789 | 9 |
| Row 9 | 640.863 | 116.6609274 | 10 | 410.877778 | 122.351 | 9 |
| Row 10 | 660.17 | 126.9402791 | 10 | 437.178889 | 135.412 | 9 |

| **ANOVA table** | **SS** | **DF** | **MS** | **F (DFn, DFd)** | **P value** | **% of total variation** |
| --- | --- | --- | --- | --- | --- | --- |
| stim number x genotype | 222828 | 9 | 24759 | F (9, 153)  = 0.8606 | P=0.5619 | 0.3276 |
| stim number | 24885535 | 9 | 2765059 | F (9, 153)  = 96.12 | P<0.0001 | 36.58 |
| genotype | 3922498 | 1 | 3922498 | F (1, 17) =  1.936 | P=0.1821 | 5.766 |
|  |  |  |  | F (17, |  |  |
| cell | 34446215 | 17 | 2026248 | 153) = | P<0.0001 | 50.64 |
|  |  |  |  | 70.44 |  |  |

| **Šídák's multiple comparisons test** | **Predicted (LS) mean diff.** | | **95.00% CI of diff.** | **Adjusted P Value** |
| --- | --- | --- | --- | --- |
| EphB2 KO +AAV-Efnb1 - EphB2 KO +AAV-TdTomato | |  |  |  |
| Row 1 | 258.6 | | -364.4 to 881.6 | 0.9362 |
| Row 2 | 428.5 | | -194.5 to 1052 | 0.4181 |
| Row 3 | 365.5 | | -257.5 to 988.5 | 0.6431 |
| Row 4 | 351 | | -272.0 to 974.0 | 0.6946 |
| Row 5 | 304.7 | | -318.3 to 927.7 | 0.8396 |
| Row 6 | 220.1 | | -402.9 to 843.1 | 0.9781 |
| Row 7 | 228.2 | | -394.8 to 851.2 | 0.9718 |
| Row 8 | 268 | | -355.0 to 891.0 | 0.9209 |
| Row 9 | 230 | | -393.0 to 853.0 | 0.9703 |
| Row 10 | 223 | | -400.0 to 846.0 | 0.976 |

| **Test details** | **Predicted (LS)**  **mean 1** | | **Predicted (LS)**  **mean 2** | **Predicted (LS)**  **mean diff.** | **SE of diff.** | **N1** | **N2** | **t** | **DF** |
| --- | --- | --- | --- | --- | --- | --- | --- | --- | --- |
| EphB2 KO +AAV-Efnb1 -  EphB2 KO +AAV-TdTomato | |  |  |  |  |  |  |  |  |
| Row 1 | 1852 | | 1594 | 258.6 | 219.6 | 10 | 9 | 1.178 | 170 |
| Row 2 | 1355 | | 926.9 | 428.5 | 219.6 | 10 | 9 | 1.951 | 170 |
| Row 3 | 983.4 | | 617.9 | 365.5 | 219.6 | 10 | 9 | 1.664 | 170 |
| Row 4 | 869.4 | | 518.3 | 351 | 219.6 | 10 | 9 | 1.598 | 170 |
| Row 5 | 775.4 | | 470.8 | 304.7 | 219.6 | 10 | 9 | 1.387 | 170 |
| Row 6 | 685.9 | | 465.8 | 220.1 | 219.6 | 10 | 9 | 1.002 | 170 |
| Row 7 | 661 | | 432.7 | 228.2 | 219.6 | 10 | 9 | 1.039 | 170 |
| Row 8 | 696.1 | | 428.1 | 268 | 219.6 | 10 | 9 | 1.22 | 170 |
| Row 9 | 640.9 | | 410.9 | 230 | 219.6 | 10 | 9 | 1.047 | 170 |
| Row 10 | 660.2 | | 437.2 | 223 | 219.6 | 10 | 9 | 1.015 | 170 |

Extended Data Fig. 7F

|  | **EphB2 KO +AAV-Efnb1** | | | **EphB2 KO +AAV-TdTomato** | | |
| --- | --- | --- | --- | --- | --- | --- |
|  | **Mean** | **SEM** | **N** | **Mean** | **SEM** | **N** |
| Row 1 | 0.702358474 | 0.040393587 | 10 | 0.53998151 | 0.05442 | 9 |
| Row 2 | 0.494925167 | 0.045471594 | 10 | 0.35374677 | 0.04976 | 9 |
| Row 3 | 0.440327305 | 0.047054809 | 10 | 0.30014717 | 0.04805 | 9 |
| Row 4 | 0.393342399 | 0.041074165 | 10 | 0.27798767 | 0.04932 | 9 |
| Row 5 | 0.344195277 | 0.042730811 | 10 | 0.26997301 | 0.04441 | 9 |
| Row 6 | 0.331624046 | 0.034775498 | 10 | 0.25413667 | 0.04283 | 9 |
| Row 7 | 0.342886582 | 0.044016245 | 10 | 0.25001203 | 0.04214 | 9 |
| Row 8 | 0.321490579 | 0.038578203 | 10 | 0.23955975 | 0.04731 | 9 |
| Row 9 | 0.328291162 | 0.036327106 | 10 | 0.24262151 | 0.05065 | 9 |

| **ANOVA table** | **SS** | **DF** | **MS** | **F (DFn, DFd)** | **P value** | **% of total variation** |
| --- | --- | --- | --- | --- | --- | --- |
| stim number x genotype | 0.04086 | 8 | 0.00511 | F (8, 136) = 2.293 | P=0.0246 | 0.7776 |
| stim number | 1.822 | 8 | 0.2278 | F (2.958, 50.28) = 102.3 | P<0.0001 | 34.68 |
| genotype | 0.4965 | 1 | 0.4965 | F (1, 17) = 3.294 | P=0.0872 | 9.448 |
| cell | 2.562 | 17 | 0.1507 | F (17, 136) = 67.67 | P<0.0001 | 48.76 |

| **Šídák's multiple comparisons test** | **Mean Diff.** | | **95.00% CI of diff.** | **Adjusted P Value** |
| --- | --- | --- | --- | --- |
| EphB2 KO +AAV-Efnb1 - EphB2  KO +AAV-TdTomato | |  |  |  |
| Row 1 | 0.1624 | | -0.05573 to 0.3805 | 0.2392 |
| Row 2 | 0.1412 | | -0.07270 to 0.3551 | 0.3808 |
| Row 3 | 0.1402 | | -0.07274 to 0.3531 | 0.3853 |
| Row 4 | 0.1154 | | -0.08932 to 0.3200 | 0.5767 |
| Row 5 | 0.07422 | | -0.1210 to 0.2694 | 0.9204 |
| Row 6 | 0.07749 | | -0.09875 to 0.2537 | 0.8313 |
| Row 7 | 0.09287 | | -0.09986 to 0.2856 | 0.758 |
| Row 8 | 0.08193 | | -0.1130 to 0.2769 | 0.8633 |
| Row 9 | 0.08567 | | -0.1156 to 0.2869 | 0.8494 |

| **Test details** | **Mean 1** | | **Mean 2** | **Mean Diff.** | **SE of diff.** | **N1** | **N2** | **t** | **DF** |
| --- | --- | --- | --- | --- | --- | --- | --- | --- | --- |
| EphB2 KO +AAV-Efnb1 - EphB2  KO +AAV-TdTomato | |  |  |  |  |  |  |  |  |
| Row 1 | 0.7024 | | 0.54 | 0.1624 | 0.06777 | 10 | 9 | 2.396 | 15.15 |
| Row 2 | 0.4949 | | 0.3537 | 0.1412 | 0.06741 | 10 | 9 | 2.094 | 16.63 |
| Row 3 | 0.4403 | | 0.3001 | 0.1402 | 0.06726 | 10 | 9 | 2.084 | 16.89 |
| Row 4 | 0.3933 | | 0.278 | 0.1154 | 0.06418 | 10 | 9 | 1.797 | 16.07 |
| Row 5 | 0.3442 | | 0.27 | 0.07422 | 0.06163 | 10 | 9 | 1.204 | 16.84 |
| Row 6 | 0.3316 | | 0.2541 | 0.07749 | 0.05517 | 10 | 9 | 1.405 | 15.89 |
| Row 7 | 0.3429 | | 0.25 | 0.09287 | 0.06093 | 10 | 9 | 1.524 | 17 |
| Row 8 | 0.3215 | | 0.2396 | 0.08193 | 0.06105 | 10 | 9 | 1.342 | 15.92 |
| Row 9 | 0.3283 | | 0.2426 | 0.08567 | 0.06233 | 10 | 9 | 1.374 | 14.85 |

Extended Data Fig. 7I

|  | **Mean** | **SEM** | **N** |
| --- | --- | --- | --- |
| EphB2 KO +AAV-Efnb1 | 1 | 0.04807 | 22 |
| EphB2 KO +AAV-  TdTomato | 1.108 | 1.108 | 22 |
| Statistics | t=0.9448, df=21, p=0.3555 |  |  |
